# Supplementary material for: A gene's-eye view of sexual antagonism
Source: Proc Biol Sci. 2020 Aug 12;287(1932):20201633. doi: 10.1098/rspb.2020.1633 (PMC7575522; doi:10.1098/rspb.2020.1633)
Supplement: Supplementary Material [file rspb20201633supp1.pdf]

A gene's-eye view of sexual antagonism: Supplementary Material

Thomas J. Hitchcock[1*] & Andy Gardner[1]

1. School of Biology, University of St Andrews, St Andrews, Fife, UK, KY16 9TH;

* **Corresponding author**: E-mail: th76@st-andrews.ac.uk;

Contents

1 Summary **3**

2 Methodology **3**

2.1 General methodology . . . 3

2.2 Transmission . . . 4

2.3 Mating . . . 4

2.4 Overlapping generations . . . 5

2.4.1 Asexual reproduction . . . 5

2.4.2 Sexual reproduction . . . 7

2.4.3 Weights on survival and reproduction . . . 9

2.5 Fitness . . . 10

2.5.1 Dominance in the two sexes . . . 10

2.5.2 Magnitude of fitness effects in the two sexes . . . 10

2.5.3 Parent-of-origin effects . . . 11

2.5.4 Selection in haploids vs diploids . . . 11

2.5.5 Table of genotypic fitnesses . . . 11

2.6 Recurrence equations . . . 15

2.6.1 Genotype recursions . . . 15

2.6.2 Allele frequency recursions . . . 16

3 Results **18**

3.1 Selection on fertility effects . . . 18

3.2 Selection on fertility effects with inbreeding . . . 18

3.3 Selection on survival effects . . . 18

4 Figures **19**

5 Tables **28**

6 References **39**

1 Summary

This document contains the methodology behind, and further results pertaining to, *"A gene's-eye view of sexual antagonism"*. The mathematical methods used to generate the results and the reasoning behind certain modelling choices can be found in the section **Methodology**. The results found in the main text can be seen in the section **Results**. In addition this section also contains the invasion conditions for stronger selection regimes, as well as scenarios not discussed fully in the main text including: cytoplasmic genes, genomic imprinting, and haploid selection.

2 Methodology

We consider a series of population genetic models of sexual antagonism. We consider a single locus, and ask whether a rare mutant allele X_1 will be able to invade a population of a given resident allele X_0, and how these conditions will depend on its genomic location, and assumptions about dosage, mating scheme, and age-structure.

2.1 General methodology

We take a "gene" to mean a particular copy of a nonrecombining sequence at some locus in some individual. This gene may exist in various states of the world. It may be in a female or male, it may be of maternal-origin or paternal-origin, it may be in a juvenile or adult. We refer to the particular state of the world that a gene finds itself in as its *class*, which is analogous to *context* (*sensu* Kirkpatrick, Johnson, and Barton, 2002). A gene may adopt a particular strategy or "allele", either the mutant X_1 or the resident X_0. The mutant allele frequency in class i is notated p_i.

Each generation, genes may flow between classes. The probability that a randomly sampled gene in class i at time t came from class j at time $t-1$ is notated $\pi_{i,j}$. Whereby both i and j belong to the same set of total possible classes $i, j \in I$. As the probabilities must sum to 1, then $\sum_j \pi_{i,j} = 1$. These probabilities are the usual backwards transition probabilities found in a gene flow matrix, and thus the dominant left-eigenvector of this matrix gives us the associated class reproductive values (Taylor, 1990; Taylor, 1996).

To calculate whether the mutant allele will invade from rarity, we can construct recursion equations describing the allele frequency in class i at time t, as a function of the allele frequency in the other classes at time $t-1$, we can write out recursion equations in the following form, with the allele frequency at the next time-step given by:

$$p_i'' = \sum_j \pi_{i,j} p_j' = \sum_j \pi_{i,j} p_j w_{i,j} \tag{1}$$

where $\pi_{i,j}$ is the probability that a randomly sampled gene in class i came from class j in the previous time-step, and $w_{i,j}$ is the relative fitness in the path going from class j to class i. $w_{i,j}$ will typically be a function of the allele frequency in the different classes, and $\pi_{i,j}$ will be a function of both the allele frequency in different classes and the fitness. For the scenarios we consider here, when the mutant is vanishingly rare we can reasonably approximate the actual values of $\pi_{i,j}$ with those calculated for a

population which is monomorphic for the resident strategy $\tilde{\pi}_{i,j}$.

$$\begin{aligned} p_i'' &= \sum_j \pi_{i,j} p_j' \\ &= \sum_j (\tilde{\pi}_{i,j} + \delta\tilde{\pi}_{i,j})(p_j + \delta p_j) \\ &= \sum_j (\tilde{\pi}_{i,j} p_j + \tilde{\pi}_{i,j} \delta p_j + \delta\tilde{\pi}_{i,j} p_j + \delta\tilde{\pi}_{i,j} \delta p_j) \\ &= \sum_j \tilde{\pi}_{i,j} p_j' + O(\delta^2) \end{aligned} \tag{2}$$

Using these recursion equations we can ask when the mutant allele will be able to invade from rarity. If the $p = 0$ equilibrium point is unstable then the mutant will be able to invade. To determine the stability, we first calculate the Jacobian matrix $\mathbf{J}$, analysed when the allele is vanishingly rare in the population (Otto and Day, 2011). Each entry of the matrix is given by:

$$\mathbf{J}_{i,j} = \left.\frac{\partial p_i''}{\partial p_j}\right|_{p_i, p_j = 0} \tag{3}$$

If the leading eigenvalue of this matrix is greater than one, $\lambda_{max} > 1$ then the mutant allele will be able to invade. We can write out our eigenvalue, and thus our condition for increase, in the following form:

$$\lambda_{max} = 1 + \sum_i c_i a_i V_i > 1 \tag{4}$$

Where c_i is the class reproductive value (Fisher, 1930; Price and Smith, 1972; Grafen, 2006), a_i is the marginal fitness effect (i.e. Fisher's average effect (Fisher, 1930; Falconer, 1985)), and V_i is the genetic variance in class i (Fisher, 1918). When either selection is weak, or the mutant has low penetrance (e.g. Taylor, 1990; Seger and Stubblefield, 2002), then the variance is approximately equal in all classes. In which case we can write the condition for invasion as:

$$\sum_i c_i a_i > 0 \tag{5}$$

2.2 Transmission

The backwards transmission probabilities for the autosomal, pseudo-autosomal and X-linked cases can be seen in Figure S1.

For the mitochondria, in order to describe some of the diversity of transmission scenarios seen in nature (e.g. Birky Jr, 2001; Greiner, Sobanski, and Bock, 2015), we adopt the following scheme. We assume that individuals contain - and thus transmit- only a single mitochondrial type, but that they may inherit either parental type with a given probability. We allow this to be specific to each sex, such that males may disproportionately inherit a mitochondrial type from fathers, and females from mothers for example. This again can be seen in Figure S1.

2.3 Mating

To incorporate both haploid selection, and also avoid gene frequency change induced by assortative mating itself, we adopt the following scheme. Haploid gametes are produced, undergo selection, then a fraction ϕ of these haploid gametes are selected and preferentially pair with a gamete with the same

allele. This produces diploid genotypes with the following frequencies:

$$
\begin{aligned}
F_{11}, M_{11} = G_{11}(p_f, p_m) &= (1-\phi)p_f p_m + \phi \begin{cases} p_m & \text{if } p_f \geq p_m \\ p_f & \text{if } p_f \leq p_m \end{cases} \\
F_{01}, M_{01} = G_{01}(p_f, p_m) &= (1-\phi)(1-p_f)p_m + \phi \begin{cases} 0 & \text{if } p_f \geq p_m \\ p_m - p_f & \text{if } p_f \leq p_m \end{cases} \\
F_{10}, M_{10} = G_{10}(p_f, p_m) &= (1-\phi)p_f(1-p_m) + \phi \begin{cases} p_f - p_m & \text{if } p_f \geq p_m \\ 0 & \text{if } p_f \leq p_m \end{cases} \\
F_{00}, M_{00} = G_{00}(p_f, p_m) &= (1-\phi)(1-p_f)(1-p_m) + \phi \begin{cases} 1-p_f & \text{if } p_f \geq p_m \\ 1-p_m & \text{if } p_f \leq p_m \end{cases}
\end{aligned}
\tag{6}
$$

When selection is weak, $p_f \approx p_m \approx p$, and thus genotype frequencies can be approximated to:

$$
\begin{aligned}
F_{11} &\approx M_{11} \approx (1-\phi)p^2 + \phi p \\
F_{01} &\approx M_{01} \approx (1-\phi)(1-p)p \\
F_{10} &\approx M_{10} \approx (1-\phi)p(1-p) \\
F_{00} &\approx M_{00} \approx (1-\phi)(1-p)^2 + \phi(1-p)
\end{aligned}
\tag{7}
$$

This also has the useful property that when selection is weak, our assortative parameter ϕ, is equivalent to Wright's inbreeding coefficient (Wright, 1922), the kin-selection coefficient of genetic relatedness between the maternal-origin and paternal-origin genes (Hamilton, 1964).

2.4 Overlapping generations

> "To what extent will persons of this age, on the average, contribute to the ancestry of future generations? The question is one of some interest, since the direct action of Natural Selection must be proportional to this contribution." – (Fisher, 1930, p27)

We may also allow for overlapping generations in our analysis (e.g. Charlesworth, 1994; Caswell, 2001). Commonly, the flow of individuals through different phases of the life-cycle is described in the language of birth rates and survival probabilities. We can take these parameters and translate them into backwards transition probabilities instead, $\pi_{i,j}$, allowing us to describe our population in terms of class reproductive values, i.e. the share of a future population's ancestry that different age classes will have. These provide the correct weightings for allele-frequency changes such that asymptotic change is recovered (Fisher, 1930; Price and Smith, 1972; Taylor, 1990). Thus class reproductive value also describes the relative importance of selection on different age cohorts.

We first analyse the asexual case, extend it to sexually reproducing individuals, and then apply it to our model.

2.4.1 Asexual reproduction

Let us take a population of size n, which is growing at rate λ, and has attained a stable age distribution. There are n_a individuals of age a, and they have an effective birth rate of b_a, i.e. they produce b_a new individuals in the next census point. This may be thought of as a compound of the actual offspring

produced, and the survival of those offspring to the first census. Individuals then survive from age 1 to age a with probability l_a. This can also be written in terms of mortality rates, where μ_a is the probability of death in the interval $a-1$ to a.

$$l_a = \prod_{i=2}^{a}(1-\mu_i) \tag{8}$$

If b is the per capita birth rate of the population, then the relative size of each age class is:

$$u_a = \frac{n_a}{n} = b\frac{l_a}{\lambda^a} \tag{9}$$

Thus the probability that a gene sampled in an age 1 individual came from an age a individual in the previous census is given by:

$$\pi_{1,a} = \frac{b_a}{b}u_a = \frac{b_a l_a}{\lambda^a} \tag{10}$$

For $a > 1$, $\pi_{a,a-1} = 1$. Using these backward transition probabilities we can calculate the reproductive value of the different age-classes.

$$c_a = \sum_{i=1}^{\infty}\pi_{a,i}c_i = \pi_{a,a+1}c_{a+1} + \pi_{1,a}c_1 = c_{a+1} + \frac{b_a l_a}{\lambda^a}c_1 = c_1\sum_{i=a}^{\infty}\frac{b_i l_i}{\lambda^i} \tag{11}$$

This provides the appropriate weighting for the allele-frequency across different age classes, and thus provides a measure of the force of selection on that age class (Medawar, 1946; Medawar, 1952; Hamilton, 1966). Focusing on the newborn cohort ($a = 1$), we can also see that their reproductive value is equivalent to $1/T$, where T is the mean parental age, a classic measure of generation time (Hamilton, 1966; Charlesworth, 1994). We can see this as:

$$T = \frac{\sum_{a=1}^{\infty} a n_a b_a}{\sum_{a=1}^{\infty} n_a b_a} = \sum_{a=1}^{\infty} a\frac{b_a l_a}{\lambda^a} \tag{12}$$

$$1 = \sum_{a=1}^{\infty} c_a = \sum_{a=1}^{\infty}\left(c_1\sum_{i=a}^{\infty}\frac{b_i l_i}{\lambda^i}\right) = c_1\sum_{a=1}^{\infty}\left(\sum_{i=a}^{\infty}\frac{b_i l_i}{\lambda^i}\right) = c_1\sum_{a=1}^{\infty}a\frac{b_a l_a}{\lambda^a} = c_1 T \tag{13}$$

Equation 11 provides an expression for the reproductive value of the age a class, and thus how a change in allele frequency in that class should be weighted. This weighting is composed of two processes - survival and reproduction - which selection may act on differently.

$$c_a = c_{a,r} + c_{a,s} \tag{14}$$

The force of selection on reproduction is given by the value of the newborn individuals that class a produces. If all newborn individuals are the same, then this is simply their share in the newborn class:

$$c_{a,r} = \pi_{1,a}c_1 = c_1\frac{b_a l_a}{\lambda^a} = \frac{1}{T}\frac{b_a l_a}{\lambda^a} \tag{15}$$

The force of selection on survival is given by the value of survivors that class a produces, i.e. the age $a+1$ cohort.

$$c_{a,s} = c_{a+1} = c_1\sum_{i=a+1}^{\infty}\frac{b_i l_i}{\lambda^i} = \frac{1}{T}\sum_{i=a+1}^{\infty}\frac{b_i l_i}{\lambda^i} \tag{16}$$

We can see how these equations also match with Hamilton's expressions for the force of selection on survival and reproduction. Our equation 15 is the same as his equation 8, and our equation 16 is the same as his equation 25, except ours is relative birth rate, and his absolute (Hamilton, 1966).

As well as classes, reproductive value is often framed in terms of individuals $v_a = c_a/u_a$. Commonly, individual reproductive value is scaled such that the reproductive value of a newborn female is 1, which in the discrete time case is $v_1 = 1$ (Fisher, 1930). With the above notation:

$$\frac{v_a}{v_1} = \frac{c_a}{u_a}\frac{u_1}{c_1} = \frac{u_1}{u_a}\sum_{i=a}^{\infty}\frac{b_i l_i}{\lambda^i} = \frac{\lambda^{a-1}}{l_a}\sum_{i=a}^{\infty}\frac{b_i l_i}{\lambda^i} \tag{17}$$

This is a discrete time analogue of Hamilton's equation 22.

2.4.2 Sexual reproduction

> "If we consider the aggregate of an entire generation of such offspring it is clear that the total reproductive value of the males in this group is exactly equal to the total value of all the females, because each sex must supply half the ancestry of all future generations of the species" – (Fisher, 1930, p142)

We can introduce sexual reproduction for arbitrary ploidy in the following way. Let us now split our population into two portions, females n_f, and males n_m. Let us retain the above notation for the female specific parameters, and let us introduce κ_a for the male specific survival probabilities, and ν_a for the mortalities.

$$\kappa_a = \prod_{i=2}^{a}(1-\nu_i) \tag{18}$$

And rather than b_a for birth rate, we have m_a for the fertilisation rate at age a. We also allow individuals to vary their sex-ratio strategy as a function of age, where z_a is the fraction of males produced by females of age a, and ζ_a is the fraction of males from male fertilisations of age a. We place the following constraints on our system:

$$\begin{aligned} n_f\bar{b} = \sum_{a=1}^{\infty} n_{f,a}b_a &= \sum_{a=1}^{\infty} n_{m,a}m_a = n_m\overline{m} \\ n_f\bar{b}(1-\bar{z}) = \sum_{a=1}^{\infty} n_{f,a}b_a(1-z_a) &= \sum_{a=1}^{\infty} n_{m,a}m_a(1-z_a) = n_m\overline{m}(1-\bar{\zeta}) \\ n_f\bar{b}\bar{z} = \sum_{a=1}^{\infty} n_{f,a}b_a z_a &= \sum_{a=1}^{\infty} n_{m,a}m_a z_a = n_m\overline{m}\bar{\zeta} \end{aligned} \tag{19}$$

From this we can see that the mean maternal age for a female and male are respectively:

$$\begin{aligned} T_{f,f} =& \frac{\sum_{a=1}^{\infty} a n_{f,a}b_a(1-z_a)}{\sum_{a=1}^{\infty} n_{f,a}b_a(1-z_a)} = \sum_{a=1}^{\infty} a\frac{b_a l_a(1-z_a)}{\lambda^a} \\ T_{f,m} =& \frac{\sum_{a=1}^{\infty} a n_{f,a}b_a z_a}{\sum_{a=1}^{\infty} n_{f,a}b_a z_a} = \sum_{a=1}^{\infty} a\frac{b_a l_a z_a}{\lambda^a} \end{aligned} \tag{20}$$

And the mean paternal age for a female and male respectively are:

$$\begin{aligned} T_{m,f} =& \frac{\sum_{a=1}^{\infty} a n_{m,a}m_a(1-\zeta_a)}{\sum_{a=1}^{\infty} n_{m,a}m_a(1-\zeta_a)} = \sum_{a=1}^{\infty} a\frac{m_a \kappa_a(1-\zeta_a)}{\lambda^a} \\ T_{m,m} =& \frac{\sum_{a=1}^{\infty} a n_{m,a}m_a\zeta_a}{\sum_{a=1}^{\infty} n_{m,a}m_a\zeta_a} = \sum_{a=1}^{\infty} a\frac{m_a \kappa_a \zeta_a}{\lambda^a} \end{aligned} \tag{21}$$

To allow for arbitrary ploidy, we notate the genetic share of an individual female that a mother gets α, and the genetic share of a son that a father gets β. We now calculate the backward transition probabilities for our four cases:

$$\begin{aligned} \pi_{\{f,a\},\{f,1\}} =& \alpha\frac{b_a l_a(1-z_a)}{\lambda^a} \\ \pi_{\{f,a\},\{m,1\}} =& (1-\beta)\frac{b_a l_a z_a}{\lambda^a} \\ \pi_{\{m,a\},\{f,1\}} =& (1-\alpha)\frac{m_a \kappa_a(1-\zeta_a)}{\lambda^a} \\ \pi_{\{m,a\},\{m,1\}} =& \beta\frac{m_a \kappa_a \zeta_a}{\lambda^a} \end{aligned} \tag{22}$$

With these backward transition probabilities, we can now calculate the class reproductive values for females of age a:

$$\begin{aligned} c_{f,a} =& c_{f,a+1} + \pi_{\{f,a\},\{f,1\}}c_{f,1} + \pi_{\{f,a\},\{m,1\}}c_{m,1} \\ =& c_{f,1}\alpha\sum_{i=a}^{\infty}\frac{b_i l_i(1-z_i)}{\lambda^i} + c_{m,1}(1-\beta)\sum_{i=a}^{\infty}\frac{b_i l_i z_i}{\lambda^i} \end{aligned} \tag{23}$$

And males of age a:

$$\begin{aligned} c_{m,a} =& c_{m,a+1} + \pi_{\{m,a\},\{f,1\}} c_{f,1} + \pi_{\{m,a\},\{m,1\}} c_{m,1} \\ =& c_{f,1}(1-\alpha) \sum_{i=a}^{\infty} \frac{m_i \kappa_i (1-\zeta_i)}{\lambda^i} + c_{m,1} \beta \sum_{i=a}^{\infty} \frac{m_i \kappa_i \zeta_i}{\lambda^i} \end{aligned} \tag{24}$$

We can then write the total reproductive value of males and females in terms of the newborns, mean parental ages, and sex-specific shares in offspring.

$$\begin{aligned} c_f =& \sum_{a=1}^{\infty} c_{f,a} = c_{f,1} \alpha T_{f,f} + c_{m,1}(1-\beta) T_{f,m} \\ c_m =& \sum_{a=1}^{\infty} c_{m,a} = c_{f,1}(1-\alpha) T_{m,f} + c_{m,1} \beta T_{m,m} \end{aligned} \tag{25}$$

We can also write the total class reproductive values of males and females as:

$$\begin{aligned} c_f =& \alpha c'_{f,1} + (1-\beta) c'_{m,1} + \sum_{a=2}^{\infty} c'_{f,a} = \alpha c'_{f,1} + (1-\beta) c'_{m,1} + c'_f - c'_{f,1} \\ c_m =& (1-\alpha) c'_{f,1} + \beta c'_{m,1} + \sum_{a=2}^{\infty} c'_{m,a} = (1-\alpha) c'_{f,1} + \beta c'_{m,1} + c'_m - c'_{m,1} \end{aligned} \tag{26}$$

As the class reproductive values are constant over time. We can drop the primes and rearrange to:

$$\frac{c_{f,1}}{c_{m,1}} = \frac{1-\beta}{1-\alpha} \tag{27}$$

Under diploidy ($\alpha = 1/2, \beta = 1/2$), this means that the class reproductive value of newborn males and females is equal:

$$\frac{c_{f,1}}{c_{m,1}} = \frac{1/2}{1/2} = 1 \tag{28}$$

It is this constraint - that the reproductive value of the newborn females is equal to that of the newborn males - which underpins the classical sex-ratio argument that a parent should invest in the rarer sex (Fisher, 1930; Edwards, 1998). Thus we can see how this argument holds even with overlapping generations (Goodman, 1982; Grafen, 2014).

Similarly, we can recover results for haplodiploidy/X-chromosomes ($\alpha = 1/2, \beta = 0$) as a special case of equation 27:

$$\frac{c_{f,1}}{c_{m,1}} = \frac{1}{1/2} = 2 \tag{29}$$

Once again, it is this constraint that underpins sex-ratio arguments relating to haplodiploids. We can again see how this is remains under age-structure (Gardner, 2014).

Putting together equations 25 and 27, along with the constraint that $c_f + c_m = 1$, we can write the class reproductive values of newborns as so:

$$\begin{aligned} c_{f,1} &= (1-\beta)/\overline{T} \\ c_{m,1} &= (1-\alpha)/\overline{T} \end{aligned} \tag{30}$$

Where:

$$\overline{T} = \alpha(1-\beta) T_{f,f} + (1-\alpha)(1-\beta) T_{f,m} + (1-\alpha)(1-\beta) T_{m,f} + (1-\alpha)\beta T_{m,m} \tag{31}$$

And so we can express the class reproductive values for males and females as a whole cohort:

$$\frac{c_f}{c_m} = \left(\frac{1-\beta}{1-\alpha}\right) \left(\frac{\alpha T_{f,f} + (1-\alpha) T_{f,m}}{(1-\beta) T_{m,f} + \beta T_{m,m}}\right) \tag{32}$$

Once again, we can recover the results for autosomes as a special case ($\alpha = 1/2, \beta = 1/2$) (Grafen, 2014):

$$\frac{c_f}{c_m} = \frac{T_{f,f} + T_{f,m}}{T_{m,f} + T_{m,m}} = \frac{T_f}{T_m} \tag{33}$$

And extend it to other inheritance systems, e.g. haplodiploidy ($\alpha = 1/2, \beta = 0$):

$$\frac{c_f}{c_m} = 2\left(\frac{T_{f,f} + T_{f,m}}{2T_{m,f}}\right) = 2\frac{T_f}{T_m} \tag{34}$$

2.4.3 Weights on survival and reproduction

> "From an individual's point of view, survival must be weighted by the individual's reproductive value in the next time period. Current fecundity must be weighted by the reproductive value of offspring in the next time period"– (Frank, 1998, p171)

As with the asexual case, in the sexual case, for each age class, the force of selection upon fecundity relative to survival effects will be given by the relative value of the survivors produced, compared to the newborns created. For our model, we assume that individuals do not undergo senescence, and thus fecundity, sex-ratio strategy, and mortality rates remain constant with respect to age. As a consequence, we need not track each age-class separately, but can combine them instead into a single class, of which a fraction of the next generation will come from survival, and a fraction from reproduction.

We allow for sex-specific mortality, with the probability of survival each generation being $1-\mu$ for females, and $1-\nu$ for males. The fraction of individuals that came through reproduction is simply n_1/n, and the fraction through survival is $\sum_{a=2}^{\infty} n_a/n = 1 - n_1/n$.

$$\begin{aligned}
\frac{n_{f,1}}{n_f} &= \frac{n_f\lambda^{-1}b(1-z)}{n_f\lambda^{-1}b(1-z) + n_f\lambda^{-1}(1-\mu)} = \frac{b(1-z)}{b(1-z)+(1-\mu)} = \frac{\lambda-(1-\mu)}{\lambda} \\
\frac{n_{m,1}}{n_m} &= \frac{n_m\lambda^{-1}bz}{n_m\lambda^{-1}bz + n_m\lambda^{-1}(1-\nu)} = \frac{bz}{bz+(1-\nu)} = \frac{\lambda-(1-\nu)}{\lambda}
\end{aligned} \tag{35}$$

And thus the fraction of individuals who came from survival in the previous generation is given by:

$$\begin{aligned}
\sum_{a=2}^{\infty}\frac{n_{f,a}}{n_f} &= 1 - \frac{\lambda-(1-\mu)}{\lambda} = \frac{1-\mu}{\lambda} \\
\sum_{a=2}^{\infty}\frac{n_{m,a}}{n_m} &= 1 - \frac{\lambda-(1-\nu)}{\lambda} = \frac{1-\nu}{\lambda}
\end{aligned} \tag{36}$$

When the population is constant in size, then the fraction of newborns and survivors is simply μ and $1-\mu$ for females, and ν and $1-\nu$ for males.

As with above, the weights on survival and reproduction can be written in terms of class reproductive values. Weights on reproduction are given by the class reproductive values of the newborn individuals, and the relative shares that males and females have in them:

$$\begin{aligned}
\frac{c_{f,r}}{c_{m,r}} &= \frac{\alpha c_{f,1} + (1-\beta)c_{m,1}}{(1-\alpha)c_{f,1} + \beta c_{m,1}} \\
&= \frac{\alpha(1-\beta) + (1-\beta)(1-\alpha)}{(1-\alpha)(1-\beta) + \beta(1-\alpha)}
\end{aligned} \tag{37}$$

For diploidy ($\alpha = 1/2, \beta = 1/2$) this simplifies to 1, and for haplodiploidy/X chromosomes ($\alpha = 1/2, \beta = 0$) this simplifies to 2. Weights on survival are given by the reproductive value of the surviving males and females.

$$\begin{aligned}
\frac{c_{f,s}}{c_{m,s}} &= \frac{\sum_{a=1}^{\infty} c_{f,a+1}}{\sum_{a=1}^{\infty} c_{m,a+1}} = \frac{c_f - c_{f,1}}{c_m - c_{m,1}} \\
&= \left(\frac{1-\beta}{1-\alpha}\right)\left(\frac{\alpha T_{ff} + (1-\alpha)T_{fm} - 1}{(1-\beta)T_{mf} + \beta T_{mm} - 1}\right) \\
&= \left(\frac{1-\beta}{1-\alpha}\right)\left(\frac{T_f - 1}{T_m - 1}\right)
\end{aligned} \tag{38}$$

In our specific model, outlined above, the mean parental ages are:

$$T_f = \sum_{a=1}^{\infty} a \frac{l_a}{\lambda^a} b = a \frac{(1-\mu)^{a-1}}{\lambda^a} = \frac{\lambda}{\lambda - (1-\mu)} \tag{39}$$

$$T_m = \sum_{a=1}^{\infty} a \frac{\kappa_a}{\lambda^a} m = a \frac{(1-\nu)^{a-1}}{\lambda^a} = \frac{\lambda}{\lambda - (1-\nu)} \tag{40}$$

Which in our specific model means the relative weighting on survival simplifies under diploidy ($\alpha = 1/2, \beta = 1/2$) to:

$$\frac{c_{f,s}}{c_{m,s}} = \left(\frac{1-\mu}{1-\nu}\right)\left(\frac{\lambda-(1-\nu)}{\lambda-(1-\mu)}\right) \tag{41}$$

And for haplodiploidy/X-chromosomes ($\alpha = 1/2, \beta = 0$):

$$\frac{c_{f,s}}{c_{m,s}} = 2\left(\frac{1-\mu}{1-\nu}\right)\left(\frac{\lambda-(1-\nu)}{\lambda-(1-\mu)}\right) \tag{42}$$

2.5 Fitness

In choosing the fitness scheme a number of assumptions must be made. Here we explain the reasoning behind the different assumptions. The fitness scheme itself can be seen in Table S2.

2.5.1 Dominance in the two sexes

The dominance coefficient h, typically scales a fitness effect between the two homozygote states. If gene effects are purely additive then $h = 1/2$. In previous models (e.g. Rice, 1984), it has been assumed that dominance is equivalent, or at least comparable, in the two sexes. In comparison, a series of more recent models have instead assumed reversals of dominance between the two sexes (Fry, 2010; Jordan and Charlesworth, 2012; Patten, 2019). Much of this debate appears centred round phenotypic vs fitness conceptions of the work the dominance coefficient is doing. If phenotypic, then the dominance coefficient would be expected due to the non-linearities in the allelic effect on the phenotype. If the gene is acting on a similar phenotype, and through a similar pathway in the two sexes, then it would make sense that dominance would be similar in the two sexes. In contrast, if we take a fitness view, where by the homozygote genotype moves amounts S and T in fitness space. Then, due to the expected non-linearities in the fitness landscape, the dominance coefficients may be different. In particular, it has been argued that if they are moving in opposite directions with respect to their respective optima then reversals of dominance may be expected. Discussion of these points can be found in (Fry, 2010, Patten, 2019, Connallon and Chenoweth, 2019). To incorporate both of these we consider both 'equal dominance' $h_f = h_m = h$, and 'reversals of dominance' $h_f = 1 - h_m = h$ scenarios.

2.5.2 Magnitude of fitness effects in the two sexes

A second point revolves around how to compare the fitness effect in a heterozygote/homozygote to that in a hemizygote. Typically, it is assumed that the fitness effects in the hemizygote/homozygote are comparable. However, this implicitly assumes an averaging rather than an adding view of gene effects (Frank, 2003, Gardner, 2012). In general, there is not a clear theoretical argument as to which approach should be taken. Empirically, dosage balancing mechanisms, such as X-inactivation or increased gene expression in one sex, may justify an averaging rather than adding approach, as the

amount of gene product in comparison to the autosomes will be the same in the two sexes (Mank, 2013; Gu and Walters, 2017; Muyle, Shearn, and Marais, 2017).

However, this may not hold if a variant's effects are due to absolute physical copies of the gene, or if dosage balancing mechanisms do not ensure that the amount of mutant gene product is equivalent between the homozygote and the hemizygote. In these cases, the hemizygote may be regarded as more comparable to the heterozygote - an adding approach. In reality, it is likely that the extent of dosage balance - which we notate γ - will scale between these two extremes.

2.5.3 Parent-of-origin effects

Finally, genes may also have parent-of-origin effects (Ferguson-Smith, 2011). The best understood form of this is genomic imprinting, whereby a gene's level of expression depends on its parent of origin, and usually involves the silencing of a gene originating from one parent and the expression of its homologue originating from the other. This is an important aspect to consider as various mechanisms of dosage balance on sex chromosomes rely on parent-of-origin specific mechanisms. The best understood of which is in the marsupials, where X-inactivation is always paternal (Graves, 2016). Recently, there has also been evidence for genomic imprinting as a form of dosage compensation in *Silene latifolia* (Muyle, Zemp, et al., 2018), although see Krasovec et al. (2019) for an alternative interpretation.

It has also been proposed that genomic imprinting may evolve as a consequence of sexually antagonistic selection (Day and Bonduriansky, 2004). If so, then sex-specific imprinting would be expected, i.e. daughters express their maternal-origin gene copy and sons their paternal-origin gene copy. To explore these diverse pieces of biology we consider four scenarios: A) no parent-of-origin effects, B) maternal-origin silencing, C) paternal-origin silencing, D) sex-specific imprinting. For the X chromosome, we assume that the imprinting has no impact in males, and as such the imprinting only affects females.

2.5.4 Selection in haploids vs diploids

Selection may occur during haploid as well as diploid phases (reviewed by Immler (2019)). As with the homozygote/hemizygote comparison above it is not clear whether gene effects should be added or averaged. In practice, most modellers assume averaging effects (e.g. Immler, Arnqvist, and Otto, 2012). We follow that precedent here when comparing selection in haploids and diploids.

2.5.5 Table of genotypic fitnesses

Autosomal/Pseudoautosomal		F_{00}	F_{10}	F_{01}	F_{11}	M_{00}	M_{10}	M_{01}	M_{11}	f_0	f_1	m_0	m_1
No PoO effects	M+/F-	1	$1-h_fT$	$1-h_fT$	$1-T$	1	$1+h_mS$	$1+h_mS$	$1+S$	1	1	1	1
	F+/M-	1	$1+h_fS$	$1+h_fS$	$1+S$	1	$1-h_mT$	$1-h_mT$	$1-T$	1	1	1	1
Paternal-origin silencing	M+/F-	1	$1-T$	1	$1-T$	1	$1+S$	1	$1+S$	1	1	1	1
	F+/M-	1	$1+S$	1	$1+S$	1	$1-T$	1	$1-T$	1	1	1	1
Maternal-origin silencing	M+/F-	1	1	$1-T$	$1-T$	1	1	$1+S$	$1+S$	1	1	1	1
	F+/M-	1	1	$1+S$	$1+S$	1	1	$1-T$	$1-T$	1	1	1	1
Sex-specific imprinting	M+/F-	1	$1-T$	1	$1-T$	1	1	$1+S$	$1+S$	1	1	1	1
	F+/M-	1	$1+S$	1	$1+S$	1	1	$1-T$	$1-T$	1	1	1	1
Haploid selection	M+/F-	1	1	1	1	1	1	1	1	1	$1-T$	1	$1+S$
	F+/M-	1	1	1	1	1	1	1	1	1	$1+S$	1	$1-T$

Table S1: Fitness scheme for autosomal and pseudoautosomal genes

X-linked		F_{00}	F_{10}	F_{01}	F_{11}	M_0	M_1	f_0	f_1	m_0	m_1
No PoO effects	M+	1	$1-h_fT$	$1-h_fT$	$1-T$	1	$1+(h_m+\gamma(1-h_m))S$	1	1	1	1
	F+	1	$1+h_fS$	$1+h_fS$	$1+S$	1	$1-(h_m+\gamma(1-h_m))T$	1	1	1	1
Paternal-origin silencing	M+	1	$1-T$	1	$1-T$	1	$1+S$	1	1	1	1
	F+	1	$1+S$	1	$1+S$	1	$1-T$	1	1	1	1
Maternal-origin silencing	M+	1	1	$1-T$	$1-T$	1	$1+S$	1	1	1	1
	F+	1	1	$1+S$	$1+S$	1	$1-T$	1	1	1	1
Sex-specific imprinting	M+	1	$1-T$	1	$1-T$	1	$1+S$	1	1	1	1
	F+	1	$1+S$	1	$1+S$	1	$1-T$	1	1	1	1
Haploid selection	M+	1	1	1	1	1	1	1	$1-T$	1	$1+S$
	F+	1	1	1	1	1	1	1	$1+S$	1	$1-T$

Table S2: Fitness scheme for X-linked genes

Mitochondria		F_0	F_1	M_0	M_1	f_0	f_1	m_0	m_1
No PoO effects	M+	1	$1-T$	1	$1+S$	1	1	1	1
	F+	1	$1+S$	1	$1-T$	1	1	1	1
Paternal-origin silencing	M+	1	$1-T$	1	$1+S$	1	1	1	1
	F+	1	$1+S$	1	$1-T$	1	1	1	1
Maternal-origin silencing	M+	1	$1-T$	1	$1+S$	1	1	1	1
	F+	1	$1+S$	1	$1-T$	1	1	1	1
Sex-specific imprinting	M+	1	$1-T$	1	$1+S$	1	1	1	1
	F+	1	$1+S$	1	$1-T$	1	1	1	1
Haploid selection	M+	1	1	1	1	1	$1-T$	1	$1+S$
	F+	1	1	1	1	1	$1+S$	1	$1-T$

Table S3: Fitness scheme for mitochondrial genes

2.6 Recurrence equations

2.6.1 Genotype recursions

We initially write out our recurrence equations in terms of genotype frequencies. For the autosomal/pseudoautosomal case, there are 4 possible genotypes per sex. As the frequency of the genotypes within one sex must sum to 1, then we can fully describe out population with 6 variables. Similarly we need 4 variables for the X-linked case, and 2 for the mitochondrial case.

Autosomal

$$\begin{aligned}
F'_{10} =&\rho_F \left(F_{10}\frac{\psi_{F10}}{\overline{\psi}_F}\right) + (1-\rho_F)G_{10}(f'_1, m'_1)\\
F'_{01} =&\rho_F \left(F_{01}\frac{\psi_{F10}}{\overline{\psi}_F}\right) + (1-\rho_F)G_{01}(f'_1, m'_1)\\
F'_{11} =&\rho_F \left(F_{11}\frac{\psi_{F10}}{\overline{\psi}_F}\right) + (1-\rho_F)G_{11}(f'_1, m'_1)\\
M'_{10} =&\rho_M \left(M_{10}\frac{\psi_{F10}}{\overline{\psi}_F}\right) + (1-\rho_M)G_{10}(f'_1, m'_1)\\
M'_{01} =&\rho_M \left(M_{01}\frac{\psi_{F10}}{\overline{\psi}_F}\right) + (1-\rho_M)G_{01}(f'_1, m'_1)\\
M'_{11} =&\rho_M \left(M_{11}\frac{\psi_{F10}}{\overline{\psi}_F}\right) + (1-\rho_M)G_{11}(f'_1, m'_1)
\end{aligned} \tag{43}$$

Where:

$$\rho_F = \frac{1-\mu}{\lambda}; \rho_M = \frac{1-\nu}{\lambda} \tag{44}$$

$$\begin{aligned}
f'_1 &= \left(F_{01}\frac{\omega_{F01}}{2\overline{\omega}_F} + F_{10}\frac{\omega_{F10}}{2\overline{\omega}_F} + F_{11}\frac{\omega_{F11}}{\overline{\omega}_F}\right)\frac{\omega_{f1}}{\overline{\omega}_f}\\
m'_1 &= \left(M_{01}\frac{\omega_{M01}}{2\overline{\omega}_M} + M_{10}\frac{\omega_{M10}}{2\overline{\omega}_M} + M_{11}\frac{\omega_{M11}}{\overline{\omega}_M}\right)\frac{\omega_{m1}}{\overline{\omega}_m}
\end{aligned} \tag{45}$$

$$\begin{aligned}
\overline{\psi}_F =&\psi_{F00}(1-F_{01}-F_{10}-F_{11}) + \psi_{F01}F_{01} + \psi_{F10}F_{10} + \psi_{F11}F_{11}\\
\overline{\psi}_M =&\psi_{M00}(1-M_{01}-M_{10}-M_{11}) + \psi_{M01}M_{01} + \psi_{M10}M_{10} + \psi_{M11}M_{11}
\end{aligned} \tag{46}$$

$$\begin{aligned}
\overline{\omega}_F =&\omega_{F00}(1-F_{01}-F_{10}-F_{11}) + \omega_{F01}F_{01} + \omega_{F10}F_{10} + \omega_{F01}F_{11}\\
\overline{\omega}_M =&\omega_{M00}(1-M_{01}-M_{10}-M_{11}) + \omega_{M01}M_{01} + \omega_{M10}M_{10} + \omega_{M01}M_{11}
\end{aligned} \tag{47}$$

$$\begin{aligned}
\overline{\omega}_f =& \left(F_{01}\frac{\omega_{F01}}{2\overline{\omega}_F} + F_{10}\frac{\omega_{F10}}{2\overline{\omega}_F} + F_{11}\frac{\omega_{F11}}{\overline{\omega}_F}\right)\omega_{f1}\\
&+ \left(1 - F_{01}\frac{\omega_{F01}}{2\overline{\omega}_F} - F_{10}\frac{\omega_{F10}}{2\overline{\omega}_F} - F_{11}\frac{\omega_{F11}}{\overline{\omega}_F}\right)\omega_{f0}\\
\overline{\omega}_m =& \left(M_{01}\frac{\omega_{M01}}{2\overline{\omega}_M} + M_{10}\frac{\omega_{M10}}{2\overline{\omega}_M} + M_{11}\frac{\omega_{M11}}{\overline{\omega}_M}\right)\omega_{m1}\\
&+ \left(1 - M_{01}\frac{\omega_{M01}}{2\overline{\omega}_M} - M_{10}\frac{\omega_{M10}}{2\overline{\omega}_M} - M_{11}\frac{\omega_{M11}}{\overline{\omega}_M}\right)\omega_{m0}
\end{aligned} \tag{48}$$

X-linked

$$\begin{aligned}
F'_{10} =&\rho_F \left(F_{10}\frac{\psi_{F10}}{\overline{\psi}_F}\right) + (1-\rho_F)G_{10}(f'_1, m'_1)\\
F'_{01} =&\rho_F \left(F_{01}\frac{\psi_{F10}}{\overline{\psi}_F}\right) + (1-\rho_F)G_{01}(f'_1, m'_1)\\
F'_{11} =&\rho_F \left(F_{11}\frac{\psi_{F10}}{\overline{\psi}_F}\right) + (1-\rho_F)G_{11}(f'_1, m'_1)\\
M'_{1} =&\rho_M \left(M_{1}\frac{\psi_{M1}}{\overline{\psi}_M}\right) + (1-\rho_M)f'_1
\end{aligned} \tag{49}$$

$$f_1' = \left(F_{01} \frac{\omega_{F01}}{2\overline{\omega}_F} + F_{10} \frac{\omega_{F10}}{2\overline{\omega}_F} + F_{11} \frac{\omega_{F11}}{\overline{\omega}_F} \right) \frac{\omega_{f1}}{\overline{\omega}_f}$$
$$m_1' = \left(M_1 \frac{\omega_{M1}}{\overline{\omega}_M} \right) \frac{\omega_{m1}}{\overline{\omega}_m} \tag{50}$$

$$\rho_F = \frac{1-\mu}{\lambda}; \rho_M = \frac{1-\nu}{\lambda} \tag{51}$$

Where:

$$\overline{\psi}_F = \psi_{F00}(1 - F_{01} - F_{10} - F_{11}) + \psi_{F01}F_{01} + \psi_{F10}F_{10} + \psi_{F11}F_{11}$$
$$\overline{\psi}_M = \psi_{M1}M_1 + \psi_{M0}(1 - M_1) \tag{52}$$

$$\overline{\omega}_F = \omega_{F00}(1 - F_{01} - F_{10} - F_{11}) + \omega_{F01}F_{01} + \omega_{F10}F_{10} + \omega_{F01}F_{11}$$
$$\overline{\omega}_M = \omega_{M1}M_1 + \omega_{M0}(1 - M_1) \tag{53}$$

$$\overline{\omega}_f = \left(F_{01} \frac{\omega_{F01}}{2\overline{\omega}_F} + F_{10} \frac{\omega_{F10}}{2\overline{\omega}_F} + F_{11} \frac{\omega_{F11}}{\overline{\omega}_F} \right) \omega_{f1} + \left(1 - F_{01} \frac{\omega_{F01}}{2\overline{\omega}_F} - F_{10} \frac{\omega_{F10}}{2\overline{\omega}_F} - F_{11} \frac{\omega_{F11}}{\overline{\omega}_F} \right) \omega_{f0}$$
$$\overline{\omega}_m = \left(\frac{\omega_{M1}}{\overline{\omega}_M} M_1 \right) \omega_{m1} + \left(1 - \frac{\omega_{M1}}{\overline{\omega}_M} M_1 \right) \omega_{m0} \tag{54}$$

Mitochondria

$$F_1' = \rho_F \left(F_1 \frac{\psi_{F1}}{\overline{\psi}_F} \right) + (1 - \rho_F) \left(\mathbb{P}_f G_{10}(f_1', m_1') + (1 - \mathbb{P}_f) G_{01}(f_1', m_1') + G_{11}(f_1', m_1') \right)$$
$$M_1' = \rho_M \left(M_1 \frac{\psi_{M1}}{\overline{\psi}_M} \right) + (1 - \rho_M) \left((1 - \mathbb{P}_m) G_{10}(f_1', m_1') + \mathbb{P}_m G_{01}(f_1', m_1') + G_{11}(f_1', m_1') \right) \tag{55}$$

$$f_1' = \left(F_1 \frac{\omega_{F1}}{\overline{\omega}_F} \right) \frac{\omega_{f1}}{\overline{\omega}_f}$$
$$m_1' = \left(M_1 \frac{\omega_{M1}}{\overline{\omega}_M} \right) \frac{\omega_{m1}}{\overline{\omega}_m} \tag{56}$$

2.6.2 Allele frequency recursions

We can convert the above genotype recursions into allele frequency recursions using the following recipes.

Autosomal/Pseudoautosomal

$$p_{ff}' = F_{10}' + F_{11}'$$
$$p_{fm}' = F_{01}' + F_{11}'$$
$$p_{mf}' = M_{10}' + M_{11}'$$
$$p_{mm}' = M_{01}' + M_{11}'$$
$$D_f' = F_{11}' - (F_{10}' + F_{11}')(F_{01}' + F_{11}')$$
$$D_m' = M_{11}' - (M_{10}' + M_{11}')(M_{01}' + M_{11}') \tag{57}$$

Where:

$$
\begin{aligned}
F_{10} =& p_{ff}(1-p_{fm}) - \mathcal{D}_f \\
F_{01} =& (1-p_{ff})p_{fm} - \mathcal{D}_f \\
F_{11} =& p_{ff}p_{fm} + \mathcal{D}_f \\
M_{10} =& p_{mf}(1-p_{mm}) - \mathcal{D}_m \\
M_{01} =& (1-p_{mf})p_{mm} - \mathcal{D}_m \\
M_{11} =& p_{mf}(1-p_{mm}) - \mathcal{D}_m
\end{aligned}
\tag{58}
$$

X-linked

$$
\begin{aligned}
p'_{ff} =& F'_{10} + F'_{11} \\
p'_{fm} =& F'_{01} + F'_{11} \\
p'_{m} =& M'_1 \\
D'_f =& F'_{11} - (F'_{10} + F'_{11})(F'_{01} + F'_{11})
\end{aligned}
\tag{59}
$$

Where:

$$
\begin{aligned}
F_{10} =& p_{ff}(1-p_{fm}) - \mathcal{D}_f \\
F_{01} =& (1-p_{ff})p_{fm} - \mathcal{D}_f \\
F_{11} =& p_{ff}p_{fm} + \mathcal{D}_f \\
M_1 =& p_m
\end{aligned}
\tag{60}
$$

Mitochondrial

$$
\begin{aligned}
p'_f =& F'_1 \\
p'_m =& M'_1
\end{aligned}
\tag{61}
$$

Where:

$$
\begin{aligned}
F_1 =& p_f \\
M_1 =& p_m
\end{aligned}
\tag{62}
$$

3 Results

This section contains the invasion conditions for both the mutant, and the resident allele, for both selection on fertility effects, selection on survival effects, under both weak and strong selection.

3.1 Selection on fertility effects

In this section, selection acts on fertility such that the ω's of our recursion equations are as outlined in the fitness tables. There is no selection on survival and thus the ψ's in the recursion equations are all simply 1. There is no assortative mating, so $\phi = 0$.

Full invasion conditions for these different scenarios can be seen in Tables S5,S7,S9. Weak selection approximations can be see in Tables S4,S6,S8. Plots of these invasion conditions can be found in Figures S2,S3,S4,S5.

3.2 Selection on fertility effects with inbreeding

As in the previous section, selection acts on fertility such that the ω's of our recursion equations are as outlined in the fitness tables. There is no selection on survival and thus the ψ's in the recursion equations are all equal to 1. We now allow for arbitrary amounts of assortative mating. In the assortative mating scheme it needs to be known whether the allele frequency is higher in male or female gametes post-selection. For the scenarios we consider here, we can *a priori* make assumptions about when the allele frequency will be higher in males or females. We make the assumption that those scenarios where the allele is female beneficial, will generate a higher frequency in female gametes, and when it is male beneficial, there will be a higher frequency in male gametes. This was consistent with numerical iterations of our recursion equations which do not make these assumptions.

Full invasion conditions for these scenarios can be seen in Tables S9,S11,S13. Weak selection approximations can be seen in Tables S8,S10,S12. Plots of these invasion conditions can be found in Figures S6,S7,S8,S9.

3.3 Selection on survival effects

In this section, selection acts on survival such that the ψ's of our recursion equations are as outlined in the fitness tables. There is no selection on survival and thus the ω's in the recursion equations are all simply 1. There is no inbreeding, and thus $\phi = 0$.

Full invasion conditions for these scenarios can be seen in Table S15,S17,S19. Weak selection approximations can be seen in Tables S14,S16,S18. Plots of these invasion conditions can be found in Figures S10,S11,S12,S13.

4 Figures

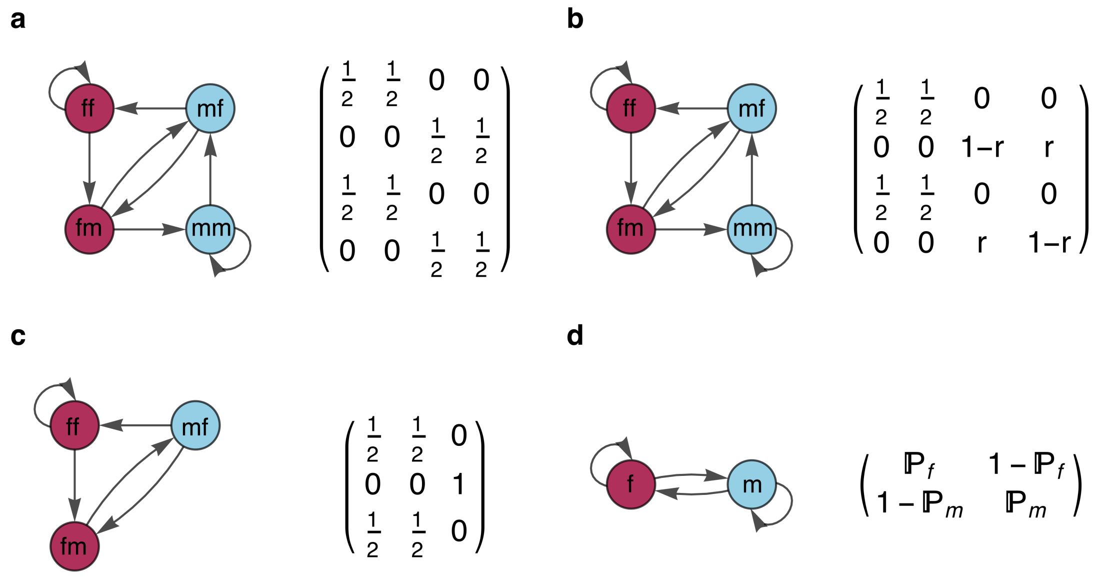

Figure S1: Gene flow matrices for the different portions of the genome we consider here. a) Autosomal genes, **b)** pseudoautosomal genes, **c)** x-linked genes, **d)** mitochondrial genes.

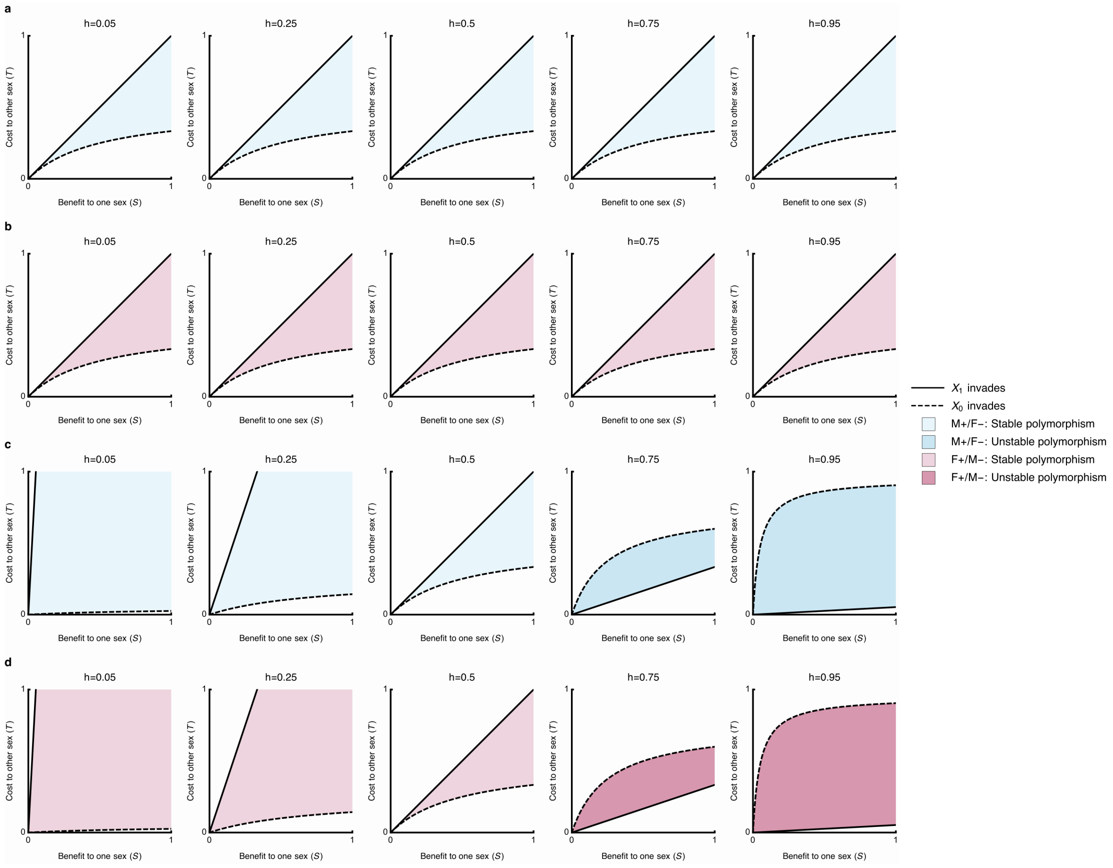

Figure S2: Invasion conditions on an autosome for the mutant allele (solid lines), and the resident allele (dashed lines), when there is complete outbreeding ($\phi = 0$), and fitness effects affect fertility. **a)** Dominance equal in the two sexes where allele is beneficial in males, costly in females. **b)** Dominance equal in the two sexes where allele is beneficial in females, costly in males. **c)** Reversals of dominance, where the allele is beneficial in males, costly in females. **d)** Reversals of dominance, where the allele is beneficial in females, costly in males.

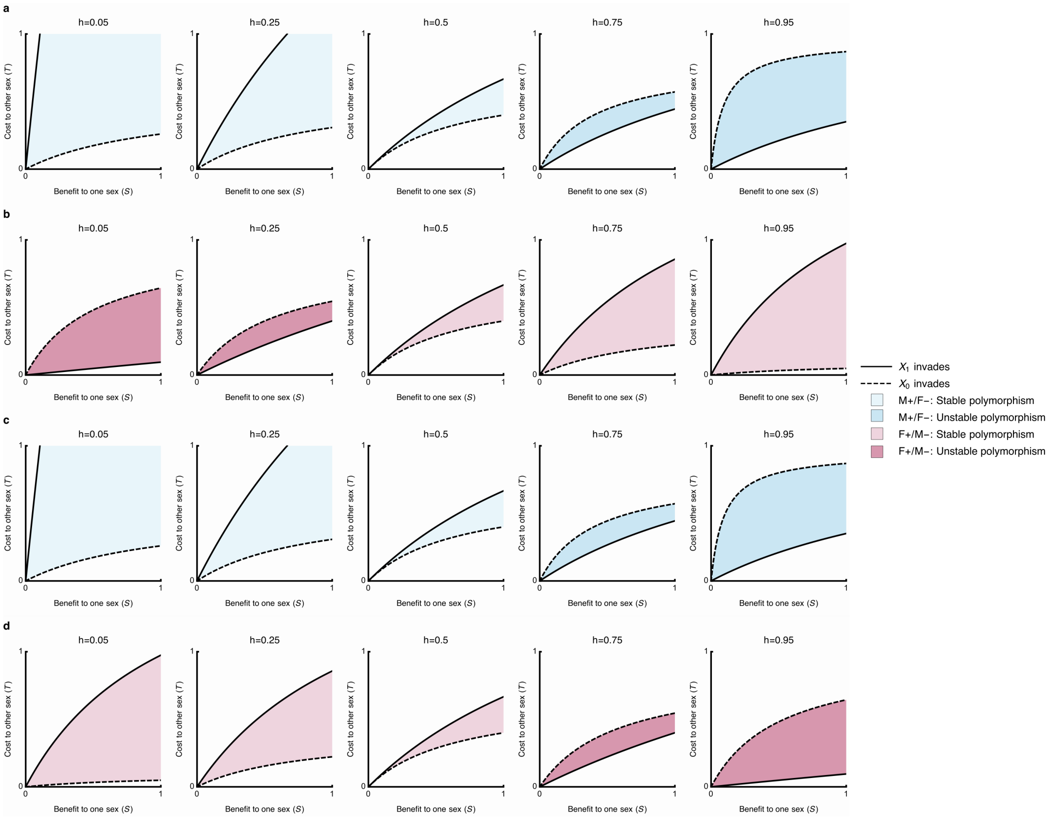

Figure S3: Invasion conditions on the X chromosome for the mutant allele (solid lines), and the resident allele (dashed lines), when there is complete outbreeding ($\phi = 0$), and fitness effects affect fertility. **a)** Dominance equal in the two sexes where allele is beneficial in males, costly in females. **b)** Dominance equal in the two sexes where allele is beneficial in females, costly in males. **c)** Reversals of dominance, where the allele is beneficial in males, costly in females. **d)** Reversals of dominance, where the allele is beneficial in females, costly in males.

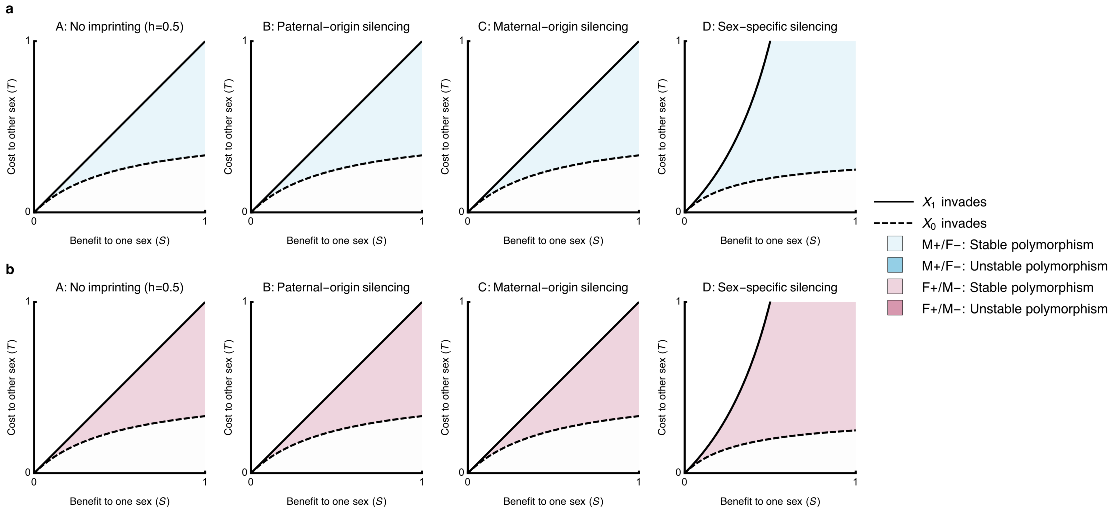

Figure S4: Invasion conditions on an autosome for the mutant allele (solid lines), and the resident allele (dashed lines),when there is complete outbreeding ($\phi = 0$), and fitness effects affect fertility. **a)** Where the allele is beneficial in males, costly in females. **b)** Where the allele is beneficial in females, costly in males.

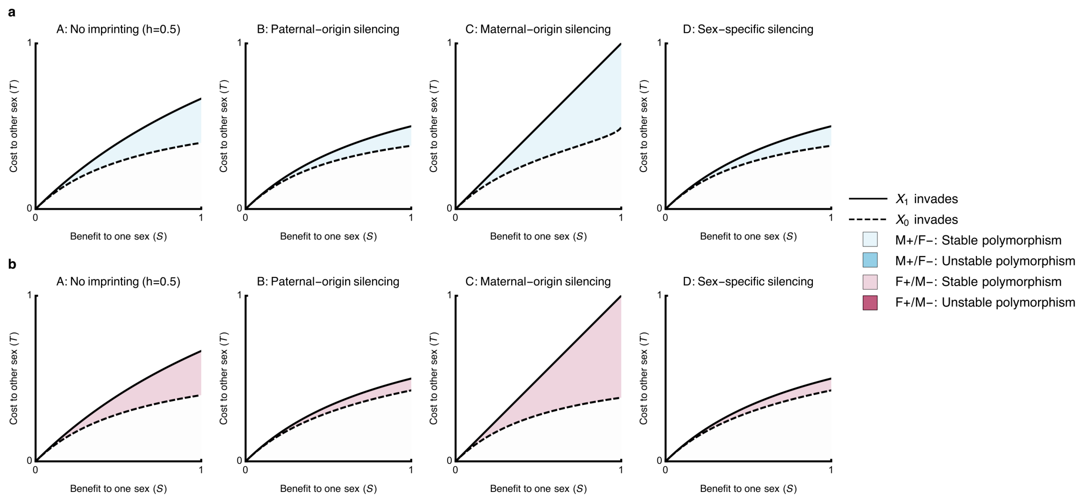

Figure S5: Invasion conditions on an X chromosome for the mutant allele (solid lines), and the resident allele (dashed lines),when there is complete outbreeding ($\phi = 0$), and fitness effects affect fertility. **a)** Where the allele is beneficial in males, costly in females. **b)** Where the allele is beneficial in females, costly in males.

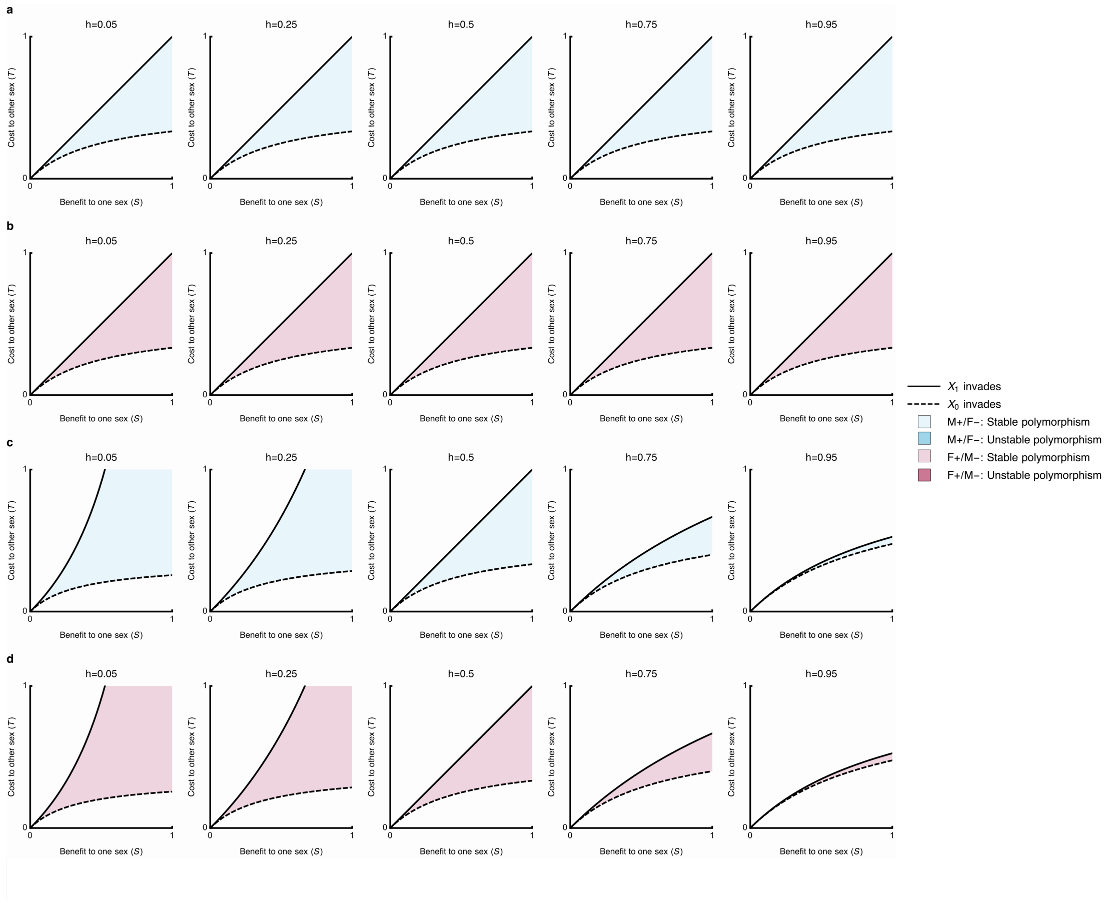

Figure S6: Invasion conditions on an autosome for the mutant allele (solid lines), and the resident allele (dashed lines), when there is complete inbreeding ($\phi = 1$), and fitness effects affect fertility. **a)** Dominance equal in the two sexes where allele is beneficial in males, costly in females. **b)** Dominance equal in the two sexes where allele is beneficial in females, costly in males. **c)** Reversals of dominance, where the allele is beneficial in males, costly in females. **d)** Reversals of dominance, where the allele is beneficial in females, costly in males.

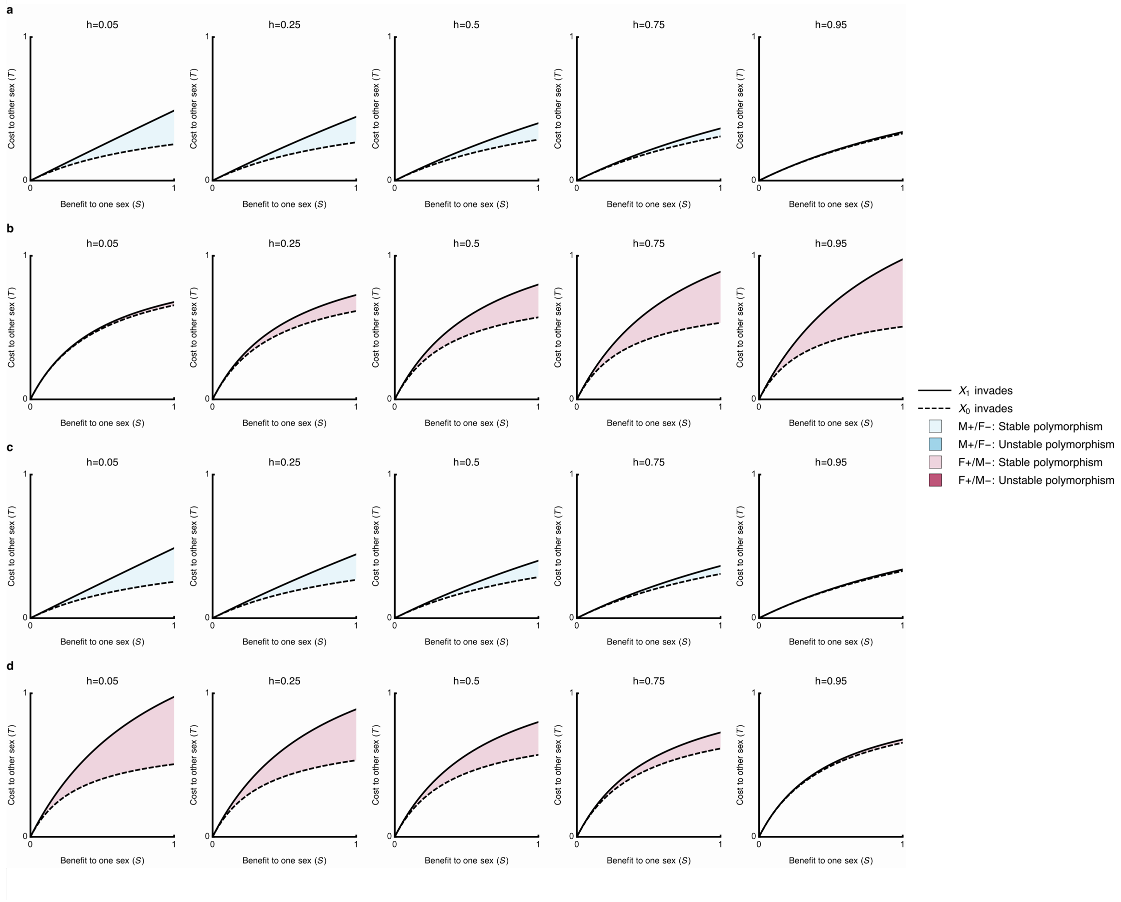

Figure S7: Invasion conditions on the X chromosome for the mutant allele (solid lines), and the resident allele (dashed lines), when there is complete inbreeding ($\phi = 1$), and fitness effects affect fertility. **a)** Dominance equal in the two sexes where allele is beneficial in males, costly in females. **b)** Dominance equal in the two sexes where allele is beneficial in females, costly in males. **c)** Reversals of dominance, where the allele is beneficial in males, costly in females. **d)** Reversals of dominance, where the allele is beneficial in females, costly in males.

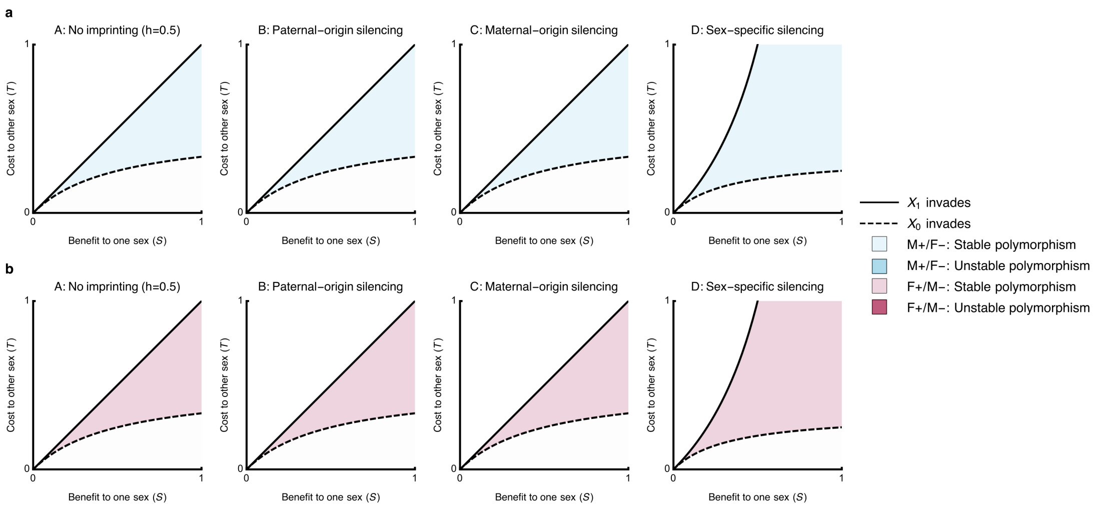

Figure S8: Invasion conditions on an autosome for the mutant allele (solid lines), and the resident allele (dashed lines),when there is complete inbreeding ($\phi = 1$), and fitness effects affect fertility. **a)** Where the allele is beneficial in males, costly in females. **b)** Where the allele is beneficial in females, costly in males.

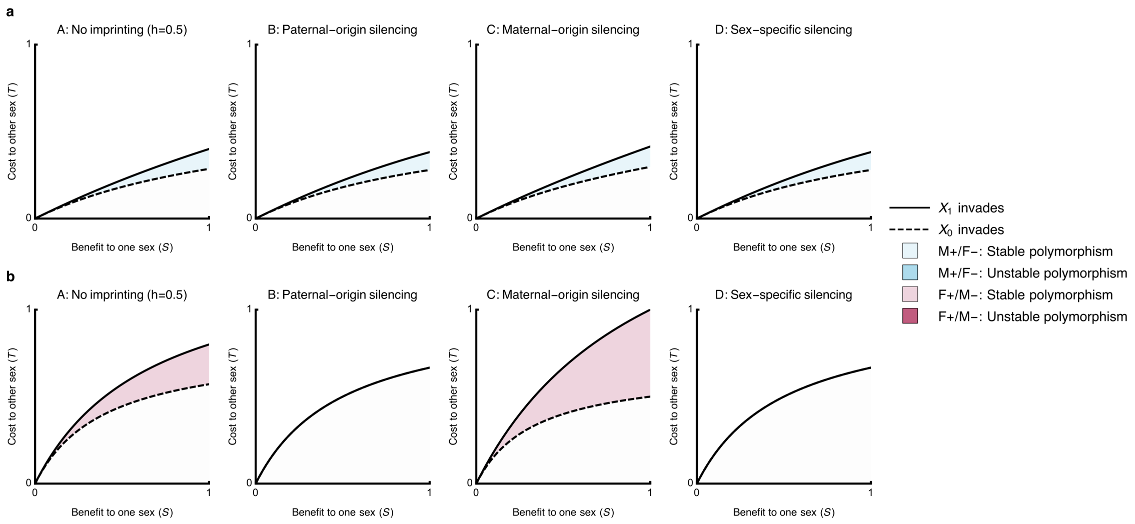

Figure S9: Invasion conditions on an X chromosome for the mutant allele (solid lines), and the resident allele (dashed lines),when there is complete inbreeding ($\phi = 1$), and fitness effects affect fertility. **a)** Where the allele is beneficial in males, costly in females. **b)** Where the allele is beneficial in females, costly in males.

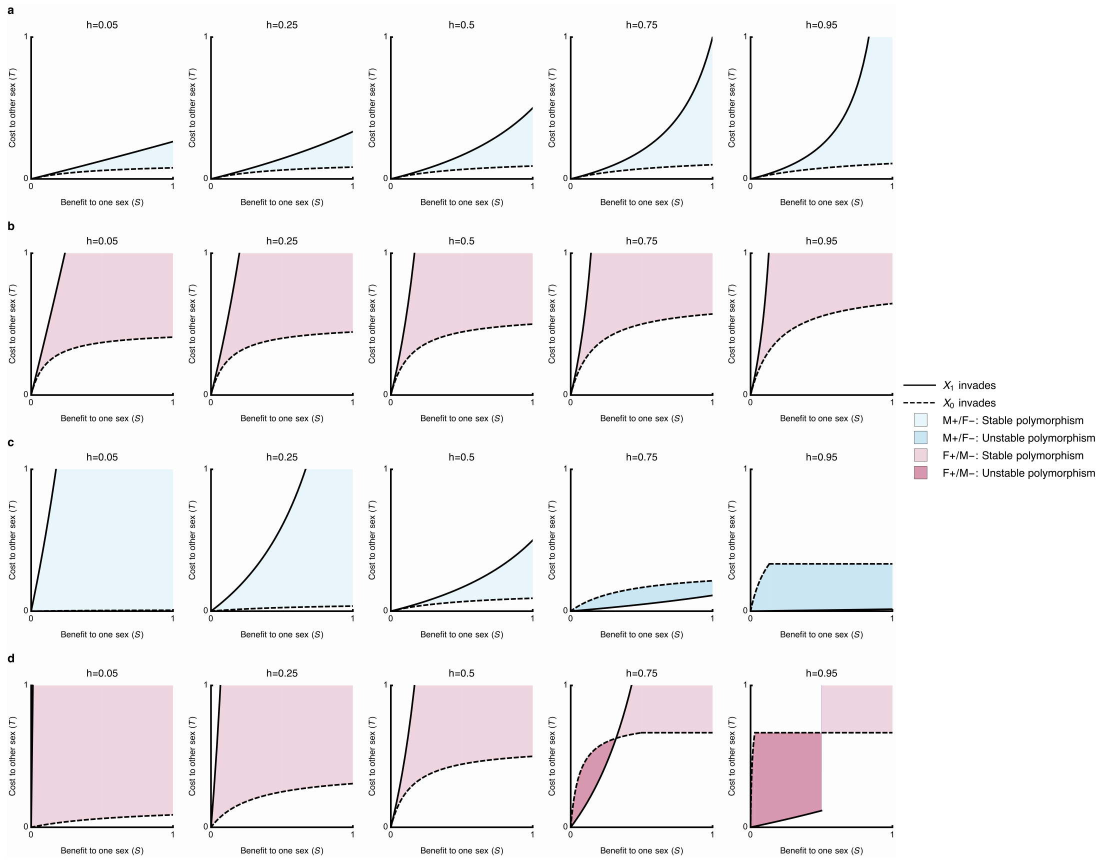

Figure S10: Invasion conditions on an autosome for the mutant allele (solid lines), and the resident allele (dashed lines), when there is complete outbreeding ($\phi = 0$), fitness effects affects survival, and there is higher male mortality ($\mu = 1/3, \nu = 2/3$). **a)** Dominance equal in the two sexes where allele is beneficial in males, costly in females. **b)** Dominance equal in the two sexes where allele is beneficial in females, costly in males. **c)** Reversals of dominance, where the allele is beneficial in males, costly in females. **d)** Reversals of dominance, where the allele is beneficial in females, costly in males.

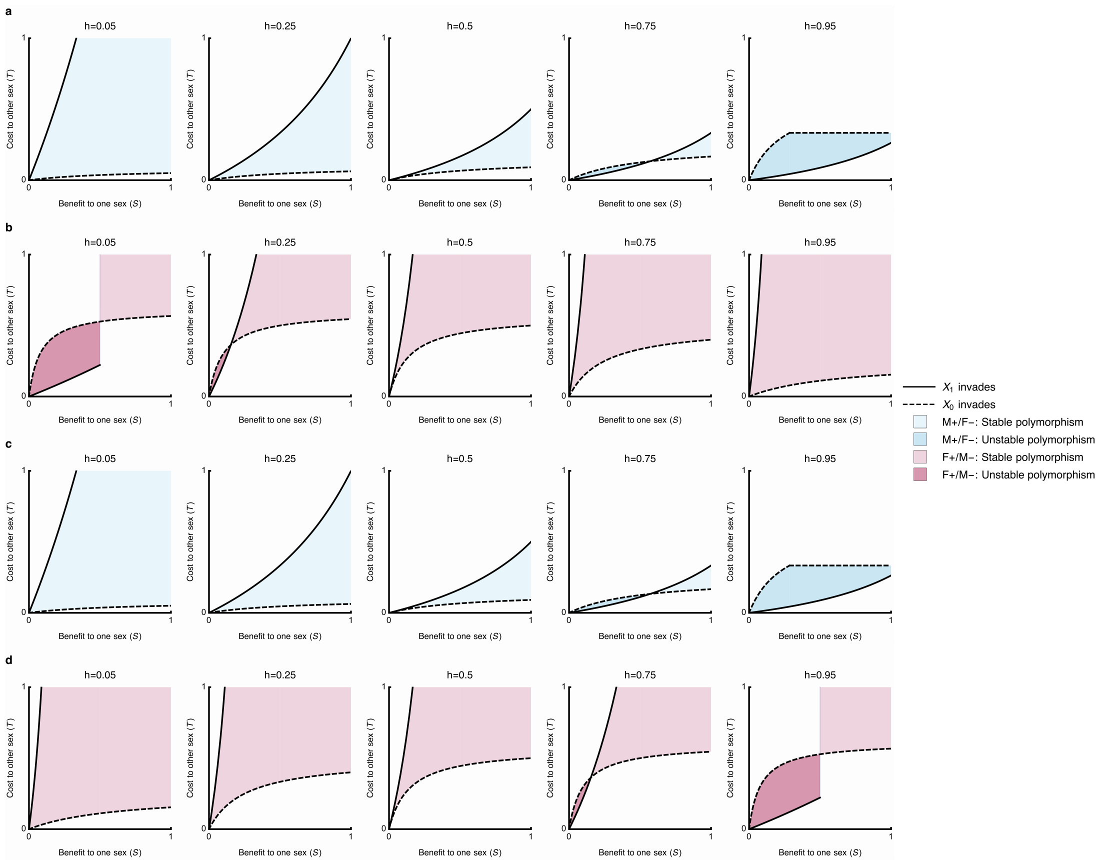

Figure S11: Invasion conditions on the X chromosome for the mutant allele (solid lines), and the resident allele (dashed lines), when there is complete outbreeding ($\phi = 0$), fitness effects affects survival, and there is higher male mortality ($\mu = 1/3, \nu = 2/3$). **a)** Dominance equal in the two sexes where allele is beneficial in males, costly in females. **b)** Dominance equal in the two sexes where allele is beneficial in females, costly in males. **c)** Reversals of dominance, where the allele is beneficial in males, costly in females. **d)** Reversals of dominance, where the allele is beneficial in females, costly in males.

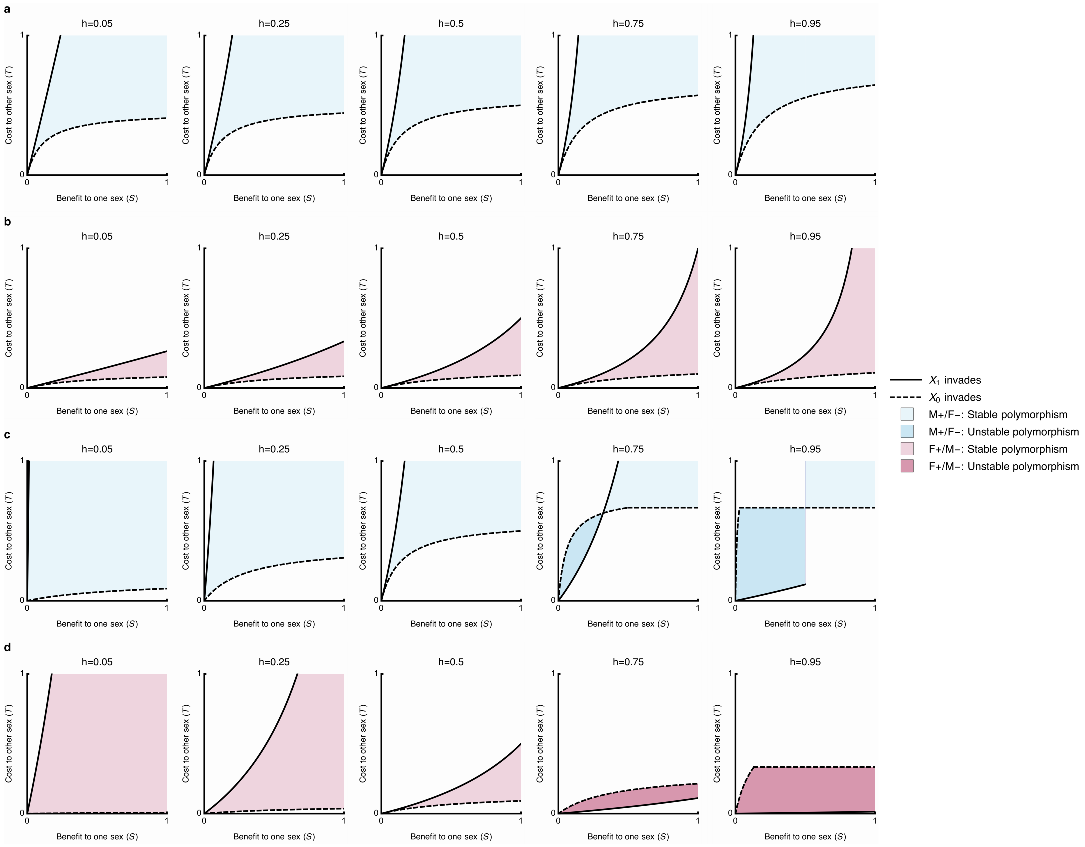

Figure S12: Invasion conditions on an autosome for the mutant allele (solid lines), and the resident allele (dashed lines), when there is complete outbreeding ($\phi = 0$), fitness effects affects survival, and there is higher female mortality ($\mu = 2/3, \nu = 1/3$). **a)** Dominance equal in the two sexes where allele is beneficial in males, costly in females. **b)** Dominance equal in the two sexes where allele is beneficial in females, costly in males. **c)** Reversals of dominance, where the allele is beneficial in males, costly in females. **d)** Reversals of dominance, where the allele is beneficial in females, costly in males.

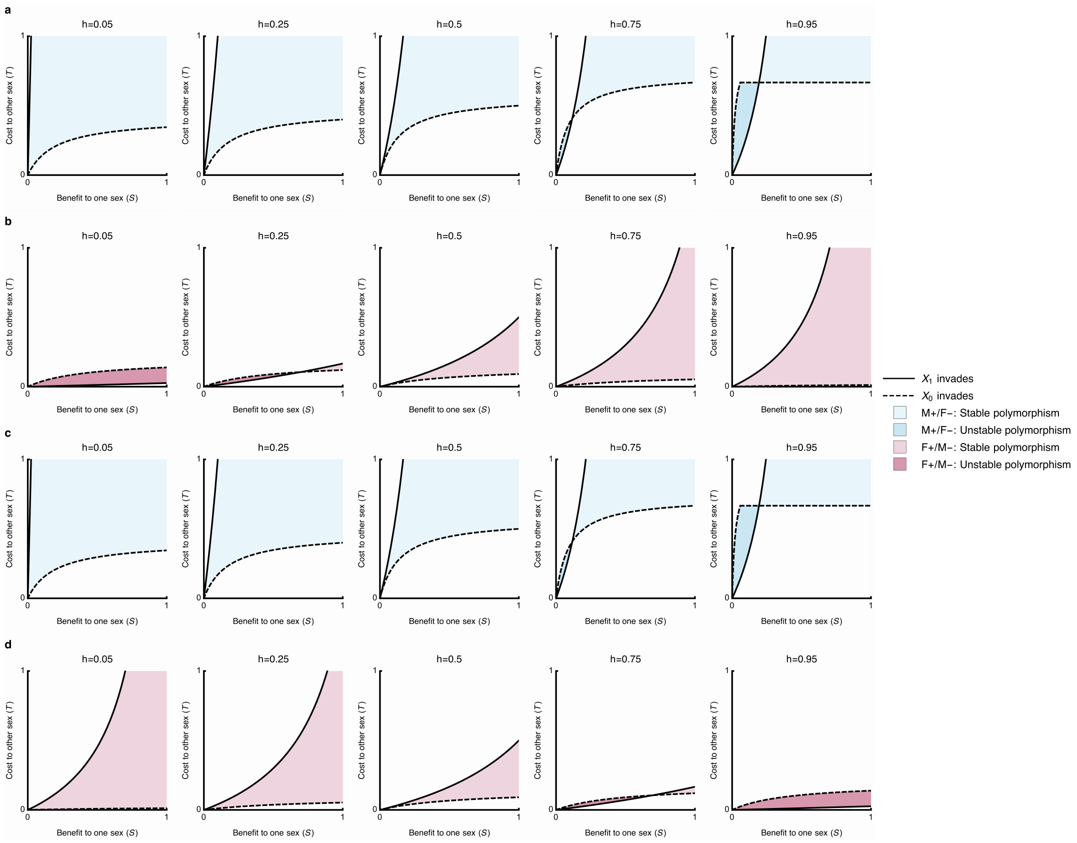

Figure S13: Invasion conditions on the X chromosome for the mutant allele (solid lines), and the resident allele (dashed lines), when there is complete outbreeding ($\phi = 0$), fitness effects affects survival, and there is higher female mortality ($\mu = 2/3, \nu = 1/3$). **a)** Dominance equal in the two sexes where allele is beneficial in males, costly in females. **b)** Dominance equal in the two sexes where allele is beneficial in females, costly in males. **c)** Reversals of dominance, where the allele is beneficial in males, costly in females. **d)** Reversals of dominance, where the allele is beneficial in females, costly in males.

5 Tables

		X_1	X_0
No PoO Effects	M+/F-	$T < \frac{h_m S}{h_f}$	$T > \frac{(h_m-1)S}{h_f-1}$
	F+/M-	$T < \frac{h_f S}{h_m}$	$T > \frac{(1-h_f)S}{1-h_m}$
All other scenarios	M+/F-	$T < S$	$T > S$
	F+/M-	$T < S$	$T > S$

Table S4: Weak selection approximations for invasion conditions for autosomal genes, with selection acting on fertility effects and full outbreeding ($\phi = 0$).

		X_1	X_0
No PoO Effects	M+/F-	$\frac{h_f T}{h_m} < S$	$\frac{T(1-h_f)}{T(2-h_f-h_m)+(1-h_m)} > S$
	F+/M-	$T < \frac{h_f S}{h_m}$	$T > \frac{(1-h_f)S}{S(2-h_f-h_m)+(1-h_m)}$
Paternal-origin silencing	M+/F-	$T < S$	$\frac{T}{1-2T} > S$
	F+/M-	$T < S$	$T > \frac{S}{2S+1}$
Maternal-origin silencing	M+/F-	$T < S$	$\frac{T}{1-2T} > S$
	F+/M-	$T < S$	$T > \frac{S}{2S+1}$
Sex-specific imprinting	M+/F-	$\frac{T}{T+1} < S$	$\frac{T}{1-3T} > S$
	F+/M-	$T < \frac{S}{1-S}$	$T > \frac{S}{3S+1}$
Haploid selection	M+/F-	$T < S$	$\frac{T}{1-2T} > S$
	F+/M-	$T < S$	$T > \frac{S}{2S+1}$

Table S5: Invasion conditions for autosomal genes, with selection acting on fertility effects and full outbreeding ($\phi = 0$).

		X_1	X_0
No PoO Effects	M+/F-	$\frac{2h_f T}{\gamma+(1-\gamma)h_m} < S$	$\frac{2(1-h_f)T}{\gamma+h_m(1-\gamma)} > S$
	F+/M-	$T < \frac{2h_f S}{\gamma+h_m(1-\gamma)}$	$T > \frac{2(1-h_f)S}{\gamma+h_m(1-\gamma)}$
All imprinting	M+/F-	$T < S$	$T > S$
	F+/M-	$T < S$	$T > S$
Haploid selection	M+/F-	$T < \frac{S}{2}$	$T > \frac{S}{2}$
	F+/M-	$T < 2S$	$T > 2S$

Table S6: Weak selection approximations of invasion conditions for X-linked genes, with selection acting on fertility effects and full outbreeding ($\phi = 0$).

		X_1	X_0
No PoO Effects	M+/F-	$\frac{2h_fT}{(1-h_fT)(\gamma+(1-\gamma)h_m)} < S$	$\frac{2(1-h_f)T}{(1+(h_f-2)T)(\gamma+h_m(1-\gamma))} > S$
	F+/M-	$T < \frac{2h_fS}{(h_fS+1)(\gamma+(1-\gamma)h_m)}$	$T > \frac{2(1-h_f)S}{(1+(2-h_f)S)(\gamma+(1-\gamma)h_m)}$
Paternal-origin silencing	M+/F-	$\frac{(1+\rho_f)T}{1+\rho_f-T} < S$	$T > \frac{(\rho_f+1)S}{\rho_f+2\rho_fS+S+1}$
	F+/M-	$\frac{(\rho_f+1)T}{1+\rho_f-T} < S$	$T > \frac{(1+\rho_f)S}{1+\rho_f+2\rho_fS+S}$
Maternal-origin silencing	M+/F-	$\frac{(1+\rho_f)T}{1+\rho_f-\rho_fT} < S$	$T > \frac{(1+\rho_f)S}{1+2S+\rho_f+S\rho_f}$
	F+/M-	$\frac{(1+\rho_f)T}{1+\rho_f-\rho_fT} < S$	$T > \frac{(1+\rho_f)S}{1+2S+\rho_f+\rho_fS}$
Sex-specific imprinting	M+/F-	$\frac{(1+\rho_f)T}{1+\rho_f-T} < S$	$T > \frac{(1+\rho_f)S}{1+S+\rho_f+2S\rho_f}$
	F+/M-	$\frac{(1+\rho_f)T}{1+\rho_f-T} < S$	$T > \frac{(1+\rho_f)S}{1+S+\rho_f+2S\rho_f}$
Haploid selection	M+/F-	$-\frac{2T}{T-1} < S$	$\frac{2T}{1-2T} > S$
	F+/M-	$T < \frac{2S}{S+1}$	$T > \frac{2S}{2S+1}$

Table S7: Invasion conditions for X-linked genes, with selection acting on fertility effects and full outbreeding ($\phi = 0$), where $\rho_f = (1-\mu)/\lambda$, and $\rho_m = (1-\nu)/\lambda$.

		X_1	X_0
All scenarios	M+/F-	$\frac{(1-\mathbb{P}_m)T}{1-\mathbb{P}_f} < S$	$\frac{(1-\mathbb{P}_m)T}{1-\mathbb{P}_f} > S$
	F+/M-	$T < \frac{(1-\mathbb{P}_m)S}{1-\mathbb{P}_f}$	$T > \frac{(1-\mathbb{P}_m)S}{1-\mathbb{P}_f}$

Table S8: Weak selection approximations for invasion conditions for mitochondrial genes, with selection acting on fertility effects.

		X_1	X_0
All scenarios	M+/F-	$\frac{(1-\mathbb{P}_m)T}{T(1-\mathbb{P}_f-\mathbb{P}_m)+(1-\mathbb{P}_f)} < S$	$\frac{(1-\mathbb{P}_m)T}{1-\mathbb{P}_f-T} > S$
	F+/M-	$T < \frac{(1-\mathbb{P}_m)S}{S(1-\mathbb{P}_f-\mathbb{P}_m)+(1-\mathbb{P}_f)}$	$T > \frac{(1-\mathbb{P}_m)S}{1-\mathbb{P}_f}$

Table S9: Invasion conditions for mitochondrial genes, with selection acting on fertility effects.

		X_1	X_0
No PoO Effects	M+/F-	$\frac{T(h_f(-\phi)+h_f+\phi)}{h_m(-\phi)+h_m+\phi} < S$	$\frac{T(h_f(\phi-1)+1)}{h_m(\phi-1)+1} > S$
	F+/M-	$T < \frac{S(h_f(-\phi)+h_f+\phi)}{h_m(-\phi)+h_m+\phi}$	$T > \frac{S(h_f(\phi-1)+1)}{h_m(\phi-1)+1}$
All other scenarios	M+/F-	$T < S$	$T > S$
	F+/M-	$T < S$	$T > S$

Table S10: Weak selection approximations for invasion conditions for autosomal genes, with selection acting on fertility effects with inbreeding.

		X_1	X_0
No PoO Effects	M+/F-	$\frac{T(h_f(-\phi)+h_f+\phi)}{-h_fT\phi+h_m(T-1)\phi+h_m+\phi} < S$	$\frac{h_fT(\phi-1)+T}{(h_f-2)T+h_m(-2T\phi+T+\phi-1)+1} > S$
	F+/M-	$T < \frac{S(h_f(-\phi)+h_f+\phi)}{-h_fS\phi+h_m(S-1)\phi+h_m+\phi}$	$T > \frac{h_fS(\phi-1)+S}{S(2h_f\phi-h_f+2)+h_m(-S+\phi-1)+1}$
Paternal-origin silencing	M+/F-	$T < S$	$\frac{T}{1-2T} > S$
	F+/M-	$T < S$	$T > \frac{S}{2S+1}$
Maternal-origin silencing	M+/F-	$T < S$	$\frac{T}{1-2T} > S$
	F+/M-	$T < S$	$T > \frac{S}{2S+1}$
Sex-specific imprinting	M+/F-	$\frac{T}{T+1} < S$	$\frac{T}{1-3T} > S$
	F+/M-	$T < \frac{S}{1-S}$	$T > \frac{S}{3S+1}$
Haploid selection	M+/F-	$T < S$	$\frac{T}{1-2T} > S$
	F+/M-	$T < S$	$T > \frac{S}{2S+1}$

Table S11: Invasion conditions for autosomal genes, with selection acting on fertility effects with inbreeding.

		X_1	X_0
No PoO Effects	M+/F-	$\frac{2T(h_f(-\phi)+h_f+\phi)}{\gamma-_m+h_m} < S$	$\frac{2T(h_f(\phi-1)+1)}{\gamma-\gamma h_m+h_m} > S$
	F+/M-	$T < -\frac{2S(h_f(-\phi)+h_f+\phi)}{(\gamma-1)h_m-\gamma}$	$T > \frac{2S(h_f(\phi-1)+1)}{\gamma-\gamma h_m+h_m}$
All imprinting	M+/F-	$T(\phi+1) < S$	$T(\phi+1) > S$
	F+/M-	$T < S(\phi+1)$	$T > S(\phi+1)$
Haploid selection	M+/F-	$2T < S$	$2T > S$
	F+/M-	$T < 2S$	$T > 1S$

Table S12: Weak selection approximations of invasion conditions for X-linked genes, with selection acting on fertility effects and inbreeding.

		X_1	X_0
No PoO Effects	M+/F-	$-\frac{2T(h_f(-\phi)+h_f+\phi)}{(h_fT-1)(\gamma-\gamma h_m+h_m)} < S$	$\frac{2T(h_f(\phi-1)+1)}{((h_f-2)T+1)(\gamma-\gamma h_m+h_m)} > S$
	F+/M-	$T < \frac{2S(h_f(-\phi)+h_f+\phi)}{((\gamma-1)h_m-\gamma)(2(h_f-1)S\phi-h_fS-1)}$	$T > \frac{2S(h_f(\phi-1)+1)}{(\gamma-\gamma h_m+h_m)(S(h_f(2\phi-1)+2)+1)}$
Paternal-origin silencing	M+/F-	$\frac{T(\phi+1)(\rho_f(T\phi+2)-T\phi+2)}{2(\rho_f-T+1)} < S$	$-\frac{T(\phi+1)((3T-2)\rho_f+T-2)}{(T(5T-6)+2)\rho_f+(T-4)T+2} > S$
	F+/M-	$T < \frac{S(\phi+1)(\rho_f+1)}{\rho_f+S\phi(\rho_f+1)+S+1}$	$T > \frac{S(\phi+1)(\rho_f(S(\phi-3)-2)-S(\phi+1)-2)}{\rho_f(S^2((\phi-2)\phi-5)-2S(\phi+3)-2)-S(S(\phi+1)^2+2(\phi+2))-2}$
Maternal-origin silencing	M+/F-	$\frac{T(\phi+1)(\rho_f(T\phi-2)-T\phi-2)}{2(T-1)\rho_f-2} < S$	$-\frac{T(\phi+1)((T-2)\rho_f+3T-2)}{((T-4)T+2)\rho_f+T(5T-6)+2} > S$
	F+/M-	$T < \frac{S(\phi+1)(\rho_f+1)}{\rho_f(S\phi+S+1)+S\phi+1}$	$T > \frac{S(\phi+1)(\rho_f(S\phi+S+2)-S(\phi-3)+2)}{\rho_f(S^2(\phi+1)^2+2S(\phi+2)+2)+S^2(5-(\phi-2)\phi)+2S(\phi+3)+2}$
Sex-specific imprinting	M+/F-	$\frac{T(\phi+1)(-\rho_f(T\phi+2)+T\phi-2)}{2(-\rho_f+T-1)} < S$	$-\frac{T(\phi+1)((3T-2)\rho_f+T-2)}{(T(5T-6)+2)\rho_f+(T-4)T+2} > S$
	F+/M-	$T < \frac{S(\phi+1)(\rho_f+1)}{\rho_f+S\phi(\rho_f+1)+S+1}$	$T > \frac{S(\phi+1)(\rho_f(S(\phi-3)-2)-S(\phi+1)-2)}{\rho_f(S^2((\phi-2)\phi-5)-2S(\phi+3)-2)-S(S(\phi+1)^2+2(\phi+2))-2}$
Haploid selection	M+/F-	$-\frac{2T}{T-1} < S$	$\frac{2T}{1-2T} > S$
	F+/M-	$T < \frac{2S}{S+1}$	$T > \frac{2S}{2S+1}$

Table S13: Invasion conditions for X-linked genes, with selection acting on fertility effects and full outbreeding ($\phi = 0$), where $\rho_f = (1-\mu)/\lambda$, and $\rho_m = (1-\nu)/\lambda$.

		X_1	X_0
No PoO Effects	M+/F-	$T < \frac{(1-\rho_f)\rho_m S(h_m(1-\phi)+\phi)}{\rho_f(1-\rho_m)(h_f(1-\phi)+\phi)}$	$T > \frac{(1-\rho_f)\rho_m S(1-h_m(1-\phi))}{\rho_f(1-\rho_m)(1-h_f(1-\phi))}$
	F+/M-	$T < \frac{\rho_f(1-\rho_m)S(h_f(1-\phi)+\phi)}{(1-\rho_f)\rho_m(h_m(1-\phi)+\phi)}$	$T > \frac{\rho_f(1-\rho_m)S(1-h_f(1-\phi))}{(1-\rho_f)\rho_m(1-h_m(1-\phi))}$
All imprinting	M+/F-	$T < \frac{(1-\rho_f)\rho_m S}{\rho_f(1-\rho_m)}$	$T > \frac{(1-\rho_f)\rho_m S}{\rho_f(1-\rho_m)}$
	F+/M-	$T < \frac{\rho_f(1-\rho_m)S}{(1-\rho_f)\rho_m}$	$T > \frac{\rho_f(1-\rho_m)S}{(1-\rho_f)\rho_m}$

Table S14: Weak selection approximations for invasion conditions for autosomal genes, with selection acting on survival effects, where $\rho_f = (1-\mu)/\lambda$, and $\rho_m = (1-\nu)/\lambda$.

		X_1	X_0
No PoO Effects	M+/F-	$\min[\frac{1-\rho_m}{\rho_m}, \frac{h_f\rho_f T - h_f\rho_f\rho_m T}{h_m\rho_m(2h_f\rho_f T-\rho_f+1)}] < S$	$T > \min[1-\rho_f, \frac{(1-h_m)(1-\rho_f)\rho_m S}{\rho_m(\rho_f(h_f(2h_m-1)S+h_f-h_m S-1)-h_m S+S)+(1-h_f)\rho_f(S+1)}]$
	F+/M-	$\min[\frac{1-\rho_f}{\rho_f}, \frac{h_m\rho_m T - h_m\rho_f\rho_m T}{h_f\rho_f(2h_m\rho_m T-\rho_m+1)}] < S$	$T > \min[1-\rho_m, \frac{(h_f-1)\rho_f(\rho_m-1)S}{2h_f h_m\rho_f\rho_m S-h_f\rho_f\rho_m S-h_f\rho_f S+h_m\rho_f\rho_m-h_m\rho_m-h_m\rho_f\rho_m S-h_m\rho_m S-\rho_f\rho_m+\rho_m+\rho_f S+\rho_m S}]$
Paternal-origin silencing	M+/F-	$\frac{\rho_f(1-\rho_m)T}{\rho_m(-\rho_f+2\rho_f T+1)} < S$	$T > \frac{(1-\rho_f)\rho_m S}{-\rho_f\rho_m+\rho_f+\rho_f S+\rho_m S}$
	F+/M-	$\frac{(1-\rho_f)\rho_m T}{\rho_f(-\rho_m+2\rho_m T+1)} < S$	$T > \frac{\rho_f(1-\rho_m)S}{-\rho_f\rho_m+\rho_m+\rho_f S+\rho_m S}$
Maternal-origin silencing	M+/F-	$\frac{\rho_f(1-\rho_m)T}{\rho_m(-\rho_f+2\rho_f T+1)} < S$	$T > \frac{(1-\rho_f)\rho_m S}{-\rho_f\rho_m+\rho_f+\rho_f S+\rho_m S}$
	F+/M-	$\frac{(1-\rho_f)\rho_m T}{\rho_f(-\rho_m+2\rho_m T+1)} < S$	$T > \frac{\rho_f(1-\rho_m)S}{-\rho_f\rho_m+\rho_m+\rho_f S+\rho_m S}$
Sex-specific imprinting	M+/F-	$\frac{\rho_f(1-\rho_m)T}{\rho_m(-\rho_f+3\rho_f T+1)} < S$	$T > \frac{(1-\rho_f)\rho_m S}{-\rho_f\rho_m+\rho_f+\rho_f\rho_m S+\rho_f S+\rho_m S}$
	F+/M-	$\frac{(1-\rho_f)\rho_m T}{\rho_f(-\rho_m+3\rho_m T+1)} < S$	$T > \frac{\rho_f(1-\rho_m)S}{-\rho_f\rho_m+\rho_m+\rho_f\rho_m S+\rho_f S+\rho_m S}$

Table S15: Invasion conditions for autosomal genes, with selection acting on survival effects and full outbreeding ($\phi = 0$), where $\rho_f = (1-\mu)/\lambda$, and $\rho_m = (1-\nu)/\lambda$.

		X_1	X_0
No PoO Effects	M+/F-	$T < \frac{(1-\rho_f)\rho_m S(\gamma+(1-\gamma)h_m)}{2\rho_f(1-\rho_m)(h_f(-\phi)+h_f+\phi)}$	$T > \frac{(1-\rho_f)\rho_m S(\gamma+(1-\gamma)h_m)}{2\rho_f(1-\rho_m)(1-h_f(1-\phi))}$
	F+/M-	$T < \frac{2\rho_f(1-\rho_m)S(h_f(1-\phi)+\phi)}{(1-\rho_f)\rho_m(\gamma(1-h_m)+h_m)}$	$T > \frac{2\rho_f(1-\rho_m)S(h_f(1-\phi)+\phi)}{(1-\rho_f)\rho_m(\gamma(1-h_m)+h_m)}$
All imprinting	M+/F-	$T < \frac{(1-\rho_f)\rho_m S}{\rho_f(1-\rho_m)(\phi+1)}$	$T > \frac{(1-\rho_f)\rho_m S}{\rho_f(1-\rho_m)(\phi+1)}$
	F+/M-	$T < \frac{\rho_f(1-\rho_m)S(\phi+1)}{(1-\rho_f)\rho_m}$	$T > \frac{\rho_f(1-\rho_m)S(\phi+1)}{(1-\rho_f)\rho_m}$

Table S16: Weak selection approximations for invasion conditions for X-linked genes, with selection acting on survival effects, where $\rho_f = (1-\mu)/\lambda$, and $\rho_m = (1-\nu)/\lambda$.

		X_1	X_0
No PoO Effects	M+/F-	$\frac{2h_f\rho_f(1-\rho_m)T}{\rho_m(\gamma+(1-\gamma)h_m)(1-\rho_f(1-2h_fT))} < S$	$T > \min[1-\rho_f, \frac{(1-\rho_f)\rho_m S(\gamma+(1-\gamma)h_m)}{\rho_f(2h_f((\gamma-1)h_m S+\rho_m-\gamma S-1)+(\gamma-1)h_m(\rho_m-2)S-2\rho_m-\gamma\rho_m S+2\gamma S+2)+\rho_m S(\gamma-\gamma h_m+h_m)}]$
	F+/M-	$\min[\frac{1-\rho_f}{\rho_f}, \frac{(1-\rho_f)\rho_m T(\gamma+(1-\gamma)h_m)}{2h_f\rho_f((1-\gamma)h_m\rho_m T-\rho_m+\gamma\rho_m T+1)}] < S$	$T > \frac{2(1-h_f)\rho_f(1-\rho_m)S}{(\gamma+(1-\gamma)h_m)(\rho_f S(-2h_f-\rho_m+2)+(1-\rho_f)\rho_m+\rho_m S)}$
Paternal-origin silencing	M+/F-	$\frac{\rho_f(\rho_f+1)(1-\rho_m)T}{\rho_m+\rho_f^2\rho_m(2T-1)} < S$	$T > \min[1-\rho_f, \frac{(1-\rho_f^2)\rho_m S}{\rho_f^2(-\rho_m+S+1)-\rho_f(\rho_m-1)(S+1)+\rho_m S}]$
	F+/M-	$\min[\frac{1-\rho_f}{\rho_f}, \frac{(1-\rho_f^2)\rho_m T}{\rho_f(\rho_f-\rho_m+\rho_f\rho_m(2T-1)+1)}] < S$	$T > \frac{\rho_f(\rho_f+1)(1-\rho_m)S}{\rho_f^2(-\rho_m)+\rho_m+S(\rho_f^2-\rho_f\rho_m+\rho_f+\rho_m)}$
Maternal-origin silencing	M+/F-	$\frac{\rho_f(1-\rho_m)T}{\rho_m-\rho_f\rho_m(1-T)} < S$	$T > \min[1-\rho_f, \frac{(1-\rho_f)\rho_m S}{\rho_f(1-\rho_m)(S+1)+\rho_m S}]$
	F+/M-	$\min[\frac{1-\rho_f}{\rho_f}, \frac{(1-\rho_f)\rho_m T}{\rho_f-\rho_f\rho_m(1-T)}] < S$	$T > \frac{\rho_f(1-\rho_m)S}{(1-\rho_f)\rho_m+\rho_f(1-\rho_m)S+\rho_m S}$
Sex-specific imprinting	M+/F-	$\frac{\rho_f(\rho_f+1)(1-\rho_m)T}{\rho_f^2\rho_m\rho_m(1-2T)} < S$	$T > 1-\rho_f, \frac{(1-\rho_f^2)\rho_m S}{\rho_f^2(-\rho_m+S+1)+\rho_f(1-\rho_m)(S+1)+\rho_m S}$
	F+/M-	$\frac{1-\rho_f}{\rho_f}, \frac{(1-\rho_f^2)\rho_m T}{\rho_f(\rho_f-\rho_m+\rho_f\rho_m(-(1-2T))+1)} < S$	$T > \frac{\rho_f(\rho_f+1)(1-\rho_m)S}{\rho_f^2(-\rho_m)+\rho_m+S(\rho_f^2-\rho_f\rho_m+\rho_f+\rho_m)}$

Table S17: Invasion conditions for X-linked genes, with selection acting on survival effects under full outbreeding ($\phi = 0$),where $\rho_f = (1-\mu)/\lambda$, and $\rho_m = (1-\nu)/\lambda$.

		X_1	X_0
No PoO Effects	M+/F-	$T < \frac{(1-\mathbb{P}_f)(1-\rho_f)\rho_m S}{(1-\mathbb{P}_m)\rho_f(1-\rho_m)}$	$T > \frac{(1-\mathbb{P}_f)(1-\rho_f)\rho_m S}{(1-\mathbb{P}_m)\rho_f(1-\rho_m)}$
	F+/M-	$T < \frac{(1-\mathbb{P}_m)\rho_f(1-\rho_m)S}{(1-\mathbb{P}_f)(1-\rho_f)\rho_m}$	$T > \frac{(1-\mathbb{P}_m)\rho_f(1-\rho_m)S}{(1-\mathbb{P}_f)(1-\rho_f)\rho_m}$

Table S18: Weak selection approximations for invasion conditions for mitochondrial genes, with selection acting on survival effects, where $\rho_f = (1-\mu)/\lambda$, and $\rho_m = (1-\nu)/\lambda$.

		X_1	X_0
All scenarios	M+/F-	$\frac{(1-\mathbb{P}_m)\rho_f(1-\rho_m)T}{\rho_m(-\mathbb{P}_f(1-\rho_f)-\rho_f(1-T)+1)} < S$	$T > \frac{(1-\mathbb{P}_f)(1-\rho_f)\rho_m S}{(1-\mathbb{P}_m)\rho_f(1-\rho_m)+\rho_f S(-(1-\mathbb{P}_f)\rho m-\mathbb{P}_m(1-\rho_m)+1)+(1-\mathbb{P}_f)\rho_m S}$
	F+/M-	$\frac{(1-\mathbb{P}_f)(1-\rho_f)\rho_m T}{\rho_f(-\mathbb{P}_m(1-\rho_m)-\rho_m(1-T)+1)} < S$	$T > \frac{(1-\mathbb{P}_m)\rho_f(1-\rho_m)S}{(1-\mathbb{P}_f)(1-\rho_f)\rho_m+S(-(1-\mathbb{P}_f)\rho_f\rho_m-\mathbb{P}_f\rho_m-\mathbb{P}_m\rho_f(1-\rho_m)+\rho_f+\rho_m)}$

Table S19: Invasion conditions for mitochondrial genes, with selection acting on survival effects, where $\rho_f = (1-\mu)/\lambda$, and $\rho_m = (1-\nu)/\lambda$.

6 References

Birky Jr, C. W. (2001). "The inheritance of genes in mitochondria and chloroplasts: laws, mechanisms, and models". In: *Annual review of genetics* 35.1, pp. 125–148.

Caswell, H. (2001). *Matrix Population Models: Construction, Analysis, and Interpretation.* Sinauer Associates.

Charlesworth, B. (1994). *Evolution in age-structured populations.* Vol. 2. Cambridge University Press Cambridge.

Connallon, T. and S. F. Chenoweth (2019). "Dominance reversals and the maintenance of genetic variation for fitness". In: *PLoS biology* 17.1, e3000118.

Day, T. and R. Bonduriansky (2004). "Intralocus sexual conflict can drive the evolution of genomic imprinting". In: *Genetics* 167.4, pp. 1537–1546.

Edwards, A. W. (1998). "Natural selection and the sex ratio: Fisher's sources". In: *The American Naturalist* 151.6, pp. 564–569.

Falconer, D. (1985). "A note on Fisher's 'average effect'and 'average excess'". In: *Genetics Research* 46.3, pp. 337–347.

Ferguson-Smith, A. C. (2011). "Genomic imprinting: the emergence of an epigenetic paradigm". In: *Nature Reviews Genetics* 12.8, pp. 565–575.

Fisher, R. A. (1918). "XV.—The Correlation between Relatives on the Supposition of Mendelian Inheritance." In: *Transactions of the Royal Society of Edinburgh* 52.2, pp. 399–433.

Fisher, R. A. (1930). *The genetical theory of natural selection.* The Clarendon Press.

Frank, S. A. (1998). *Foundations of social evolution.* Princeton University Press.

Frank, S. A. (2003). "Genetic variation of polygenic characters and the evolution of genetic degeneracy". In: *Journal of evolutionary biology* 16.1, pp. 138–142.

Fry, J. D. (2010). "The genomic location of sexually antagonistic variation: some cautionary comments". In: *Evolution: International Journal of Organic Evolution* 64.5, pp. 1510–1516.

Gardner, A. (2012). "Evolution of maternal care in diploid and haplodiploid populations". In: *Journal of evolutionary biology* 25.8, pp. 1479–1486.

Gardner, A. (2014). "Total reproductive value of juvenile females is twice that of juvenile males under X-linkage and haplodiploidy". In: *Journal of theoretical biology* 359, pp. 236–237.

Goodman, D. (1982). "Optimal life histories, optimal notation, and the value of reproductive value". In: *The American Naturalist* 119.6, pp. 803–823.

Grafen, A. (2006). "A theory of Fisher's reproductive value". In: *Journal of mathematical biology* 53.1, pp. 15–60.

Grafen, A. (2014). "Total reproductive values for females and for males in sexual diploids are not equal". In: *Journal of theoretical biology* 359, pp. 233–235.

Graves, J. A. M. (2016). "Evolution of vertebrate sex chromosomes and dosage compensation". In: *Nature Reviews Genetics* 17.1, p. 33.

Greiner, S., J. Sobanski, and R. Bock (2015). "Why are most organelle genomes transmitted maternally?" In: *Bioessays* 37.1, pp. 80–94.

Gu, L. and J. R. Walters (2017). “Evolution of sex chromosome dosage compensation in animals: a beautiful theory, undermined by facts and bedeviled by details”. In: *Genome biology and evolution* 9.9, pp. 2461–2476.

Hamilton, W. (1964). “The genetical evolution of social behaviour. I”. In: *Journal of Theoretical Biology* 7.1, pp. 1–16.

Hamilton, W. D. (1966). “The moulding of senescence by natural selection”. In: *Journal of theoretical biology* 12.1, pp. 12–45.

Immler, S. (2019). “Haploid Selection in “Diploid” Organisms”. In: *Annual Review of Ecology, Evolution, and Systematics* 50, pp. 219–236.

Immler, S., G. Arnqvist, and S. P. Otto (2012). “Ploidally antagonistic selection maintains stable genetic polymorphism”. In: *Evolution: International Journal of Organic Evolution* 66.1, pp. 55–65.

Jordan, C. Y. and D. Charlesworth (2012). “The potential for sexually antagonistic polymorphism in different genome regions”. In: *Evolution: International Journal of Organic Evolution* 66.2, pp. 505–516.

Kirkpatrick, M., T. Johnson, and N. Barton (2002). “General models of multilocus evolution”. In: *Genetics* 161.4, pp. 1727–1750.

Krasovec, M. et al. (2019). “Immediate Dosage Compensation Is Triggered by the Deletion of Y-Linked Genes in Silene latifolia”. In: *Current Biology* 29.13, pp. 2214–2221.

Mank, J. E. (2013). “Sex chromosome dosage compensation: definitely not for everyone”. In: *Trends in genetics* 29.12, pp. 677–683.

Medawar, P. B. (1946). “Old age and natural death”. In: *Modern Quarterly* 2, pp. 30–49.

Medawar, P. B. (1952). “An unsolved problem of biology”. In:

Muyle, A., R. Shearn, and G. A. Marais (2017). “The evolution of sex chromosomes and dosage compensation in plants”. In: *Genome biology and evolution* 9.3, pp. 627–645.

Muyle, A., N. Zemp, et al. (2018). “Genomic imprinting mediates dosage compensation in a young plant XY system”. In: *Nature plants* 4.9, pp. 677–680.

Otto, S. P. and T. Day (2011). *A biologist’s guide to mathematical modeling in ecology and evolution.* Princeton University Press.

Patten, M. M. (2019). “The X chromosome favors males under sexually antagonistic selection”. In: *Evolution* 73.1, pp. 84–91.

Price, G. R. and C. A. Smith (1972). “Fisher’s Malthusian parameter and reproductive value”. In: *Annals of Human Genetics* 36.1, pp. 1–7.

Rice, W. R. (1984). “Sex chromosomes and the evolution of sexual dimorphism”. In: *Evolution* 38.4, pp. 735–742.

Seger, J. and J. W. Stubblefield (2002). “Models of sex ratio evolution.” In: *Sex ratios: concepts and research methods*, pp. 1–25.

Taylor, P. D. (1990). “Allele-frequency change in a class-structured population”. In: *The American Naturalist* 135.1, pp. 95–106.

Taylor, P. D. (1996). “Inclusive fitness arguments in genetic models of behaviour”. In: *Journal of Mathematical Biology* 34.5-6, pp. 654–674.

Wright, S. (1922). “Coefficients of inbreeding and relationship”. In: *The American Naturalist* 56.645, pp. 330–338.
